# Cyclosporine-induced kidney damage was halted by sitagliptin and hesperidin via increasing Nrf2 and suppressing TNF-α, NF-κB, and Bax

**DOI:** 10.1038/s41598-024-57300-x

**Published:** 2024-03-28

**Authors:** Ahmed M. Abd-Eldayem, Sohayla Mahmoud Makram, Basim Anwar Shehata Messiha, Hanan H. Abd-Elhafeez, Mustafa Ahmed Abdel-Reheim

**Affiliations:** 1 Department of Medical Pharmacology, Faculty of Medicine, Assiut University, Assiut, Egypt; 2 Department of Pharmacology, Faculty of Medicine, Merit University, Sohâg, Egypt; 3 Department of Pharmacology and Toxicology, Faculty of Pharmacy, Beni-Suef University, Beni Suef, Egypt; 4 Department of Cell and Tissue, Faculty of Veterinary Medicine, Assiut University, Assiut, Egypt; 5 Department of Pharmaceutical Sciences, College of Pharmacy, Shaqra University, Shaqra, Saudi Arabia

**Keywords:** Cyclosporine, Sitagliptin, Hesperidin, Nephrotoxicity, Nrf2, NF-κB, Immunology, Medical research, Nephrology

## Abstract

Cyclosporine A (CsA) is employed for organ transplantation and autoimmune disorders. Nephrotoxicity is a serious side effect that hampers the therapeutic use of CsA. Hesperidin and sitagliptin were investigated for their antioxidant, anti-inflammatory, and tissue-protective properties. We aimed to investigate and compare the possible nephroprotective effects of hesperidin and sitagliptin. Male Wistar rats were utilized for induction of CsA nephrotoxicity (20 mg/kg/day, intraperitoneally for 7 days). Animals were treated with sitagliptin (10 mg/kg/day, orally for 14 days) or hesperidin (200 mg/kg/day, orally for 14 days). Blood urea, serum creatinine, albumin, cystatin-C (CYS-C), myeloperoxidase (MPO), and glucose were measured. The renal malondialdehyde (MDA), glutathione (GSH), catalase, and SOD were estimated. Renal TNF-α protein expression was evaluated. Histopathological examination and immunostaining study of Bax, Nrf-2, and NF-κB were performed. Sitagliptin or hesperidin attenuated CsA-mediated elevations of blood urea, serum creatinine, CYS-C, glucose, renal MDA, and MPO, and preserved the serum albumin, renal catalase, SOD, and GSH. They reduced the expressions of TNF-α, Bax, NF-κB, and pathological kidney damage. Nrf2 expression in the kidney was raised. Hesperidin or sitagliptin could protect the kidney against CsA through the mitigation of oxidative stress, apoptosis, and inflammation. Sitagliptin proved to be more beneficial than hesperidin.

## Introduction

Cyclosporine A (CsA), an immunosuppressive drug, prevents allograft rejection in solid organ transplantation. CsA has also been used to treat autoimmune disorders including psoriasis and rheumatoid arthritis^1–3^. However, a dangerous side effect that restricts its clinical usage is CsA nephrotoxicity^4^. Inflammatory cell infiltration, tubular shrinkage, arteriolopathy, increased immunogenicity, and tubular interstitial fibrosis are the characteristics of the described CsA-evoked nephrotoxicity^5^. Although many different factors are involved in the pathophysiology of CsA-induced nephrotoxicity, oxidative stress is crucial to the onset and advancement of this disease. The basic signs of CsA renal injury are reactive oxygen species (ROS) overruns and associated lipid peroxidation or oxidative aberrations^6^. In this regard, CsA-treated human renal mesangial cells showed an overshooting of ROS^7,8^.

Some data suggest that oxidative stress plays an important role in underlying nephrotoxicity, in particular, the superoxide (O_2_^·–^) that is the most powerful free radical generated by nicotinamide adenine dinucleotide phosphate (NADPH) oxidase-1 (NOX1) present mainly in the kidney^9,10^. Moreover, it has been observed that hypertensive patients receiving CsA medication have higher plasma hydroperoxide levels^2,10^. Additionally, LLC-PK1 tubular cells have been identified as having decreased renal antioxidants like reduced glutathione^2,11^.

The mechanisms of CsA-induced nephrotoxicity are multifactorial, with inflammatory events dominating the pathogenesis of this disorder^12,13^. Conventionally, inflammation is considered an adaptive mechanism to remove invading cells and repair damaged tissue. However, an exaggerated inflammatory response and associated production of proinflammatory cytokines, including interleukin 1 beta (IL-1β) and tumor necrosis factor-alpha (TNF-α), have been reported during the progression of kidney injury. TNF-alpha mRNA, dendritic cell count, and MHC class II antigen expression were all increased after receiving CsA treatment^13,14^.

Also, cyclosporine could induce renal tissue damage by increasing the expression of inflammatory mediators such as TNF-α and tumor growth factor (TGF)-β. The produced TNF-α stimulates dendritic cell maturation, activates the innate immune responses, and enhances the production of diverse chemokines and cytokines, as occurs in lupus nephritis ^15,16^. TNF-α is a crucial immunoregulatory and proinflammatory cytokine with pleiotropic features, including the ability to trigger an inflammatory chain reaction that results in tissue damage. One of the transcription factors whose activation has been proposed to be associated with the expression of antioxidant enzymes is nuclear factor erythroid 2-related factor-2 (Nrf-2). Moreover, Nrf2 has been reported to be one of the main regulators of the glutathione S-transferase family, which induces detoxification by increasing the cytosolic concentration of GSH. Furthermore, many studies indicated that the expression of Nrf-2 prevented excessive inflammatory responses in the renal tissue exposed to cyclosporine^17–19^. The nuclear factor-kappa B pathway has long been considered a prototypical proinflammatory signaling pathway, largely based on the role of NF-κB in the expression of proinflammatory genes including cytokines, chemokines, and adhesion molecules^20^. Also, elevated renal expressions of inducible nitric oxide synthase (iNOS) and nuclear factor kappa B (NF-κB) were noticed due to CsA administration^21^.

DPP-4 inhibitors have been shown to reduce proteinuria by reducing renal inflammatory indicators in diabetics. Numerous investigations were carried out to demonstrate the feasibility of the same effects in non-diabetic nephropathies. These investigations supported the ability of DPP-4 inhibitors to decrease proteinuria without having an impact on glucose metabolism by showing an improvement in several renal inflammatory markers^22,23^. Sitagliptin, a dipeptidyl peptidase 4 inhibitor, was the first in its class to receive approval from the US FDA in 2006 for the treatment of type 2 diabetes mellitus. In clinical trials, sitagliptin was usually well tolerated, had a negligible risk of hypoglycemia, had no discernible impact on body weight, and could be used in chronic kidney disease^24,25^. Sitagliptin might have a major role in preventing diabetic nephropathy (DN) evolution due to its anti-inflammatory and antiapoptotic properties^26,27^.

Additionally, sitagliptin improved renal functions and histopathological changes, impeded inflammation, oxidative stress, tubulointerstitial transdifferentiation, and fibrosis, and upregulated the PI3K/AKT pathway, which highlights its renoprotective effects in many rat models of diabetic nephropathy^28–30^. Sitagliptin has been shown to have nephroprotective, antioxidant, and anti-inflammatory impacts. Consequently, sitagliptin administration can limit the nephrotoxic effects of deltamethrin through its free radical-scavenging and strong antioxidant activity^31^. In addition, sitagliptin exhibited potent nephroprotective properties against the nephrotoxicity induced by methotrexate^32^, gentamicin^33^, acute IR injury^34,35^, and adenine-induced kidney disease in rats^36^.

Hesperidin, a bioflavonoid, is present in large amounts in citrus fruits. Its use has been linked to several health benefits, including antioxidant, antibacterial, antimicrobial, anti-inflammatory, and anticarcinogenic properties^37^. Hesperidin protected the renal and lung tissues of rats after ischemia–reperfusion injury^38^. Hesperidin afforded protection against sodium fluoride-induced liver, kidney, and cardiac damage in rats through antioxidant, anti-inflammatory, anti-apoptotic, and anti-autophagic mechanisms^39,40^. Similarly, hesperidin administration protected the liver and kidneys against the destructive effects of sodium arsenite^41^ and acrylamide^42^. In rats subjected to paclitaxel-induced hepatorenal toxicity, hesperidin significantly decreased mRNA expression levels of NF-κB, TNF-, IL-1, IL-6, Caspase-3, and Bax, while increasing levels of Nrf2, HO-1, and Bcl-2 in the kidney and liver^43^.

Numerous investigations using kidney injury models, such as AlCl3-induced renal damage^44^, cisplatin-induced nephrotoxicity^45^, and 5-fluorouracil-induced renal dysfunction^46^, showed the plausible renoprotective action of hesperidin. Additionally, hesperidin has been identified as a novel and promising therapeutic agent capable of modifying several cardiovascular disease risk factors (CVDs). Furthermore, in diabetic mice, hesperidin showed lower blood sugar and reduced inflammation^47^.

This study aimed to investigate the potential protective effects of sitagliptin and hesperidin against the nephrotoxicity caused by CsA and to elucidate the possible mechanisms involved. Evaluation of the nephrotoxicity and impact of the chosen therapies was done through biochemical, pathological, and protein expression investigations. Additionally, we want to compare the protective effects of sitagliptin to hesperidin as a nephroprotective tool in CsA-induced kidney injury.

## Results

### The serum levels of urea and creatinine

**Figure 1:**
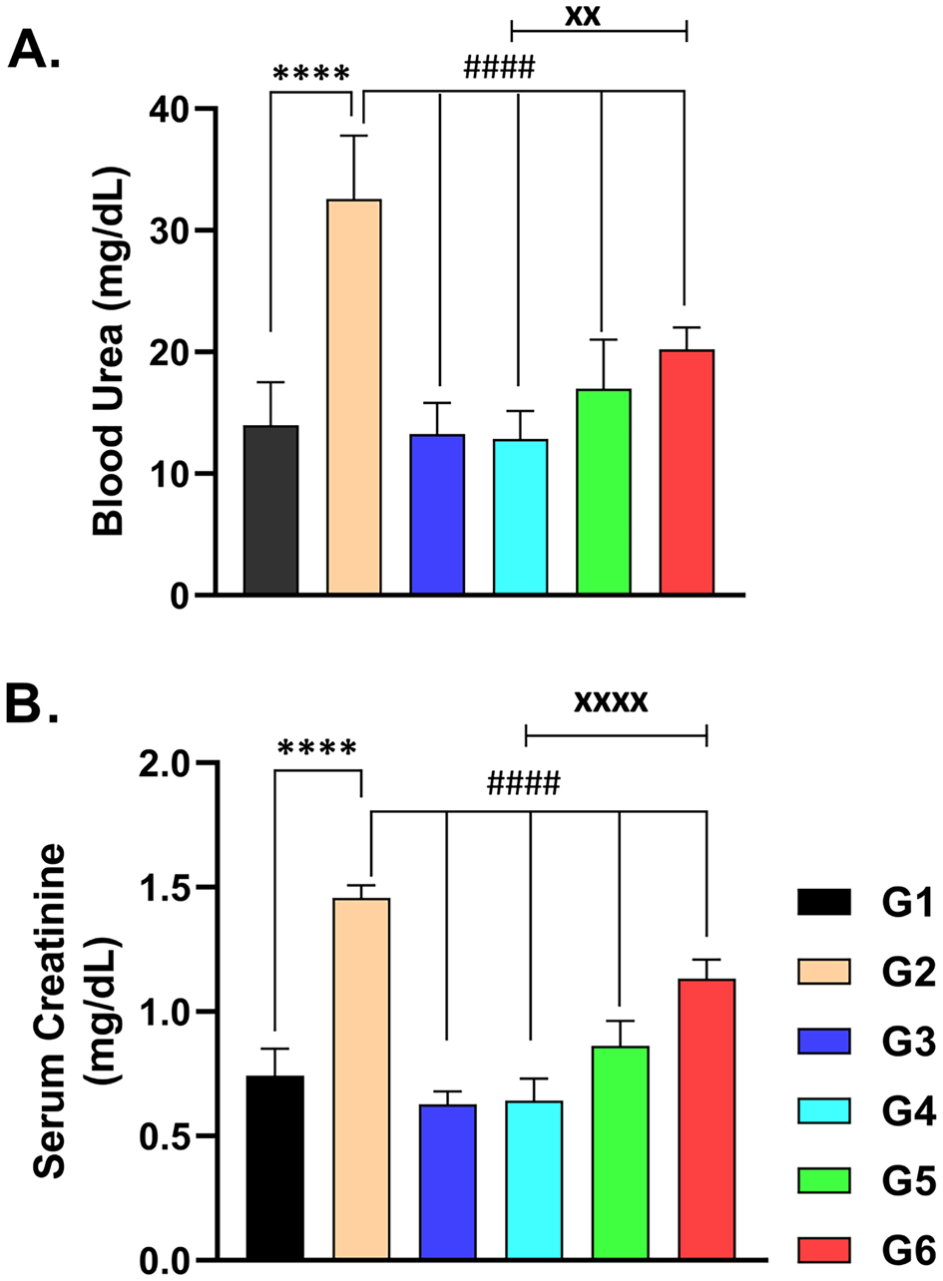
Effects of administration of cyclosporine, sitagliptin, and hesperidin on Blood urea (**A**) and serum creatinine (**B**) levels: data represented as mean ± SEM (n = 6). (G1 = Control, G2 = CsA-treated rats, G3 = Sitagliptin, G4 = Sitagliptin + CsA, G5 = Hesperidin, G6 = Hesperidin + CsA). ^****^ (*p* < 0.0001) versus G1 rats, ^####^ (*p* < 0.0001) versus G2 rats, ^xx^ (*p* < 0.01) & ^xxxx^ (*p* < 0.0001) versus G6 rats.

As shown in Fig. 1, CsA administration significantly elevated the serum levels of urea (Fig. 1A) and creatinine (Fig. 1B) as compared to normal control values (*p* < 0.0001). Using sitagliptin with CsA greatly reduced the rise in serum levels of urea and creatinine (*p* < 0.0001) as compared to CsA-treated rats. Hesperidin coadministration with CsA considerably reduced the increase in blood levels of urea and creatinine (*p* < 0.0001). Sitagliptin and hesperidin administered individually produced no significant changes in these biochemical parameters versus control rats given the vehicle. A comparative look at the figure indicates that sitagliptin treatment was greater than hesperidin in the correction of serum parameters, indicating its preservative effect on renal tissue.

### The levels of serum Albumin (ALB), blood glucose, serum myeloperoxidase (MPO), and serum cystatin-C (CYS-C)

**Figure 2:**
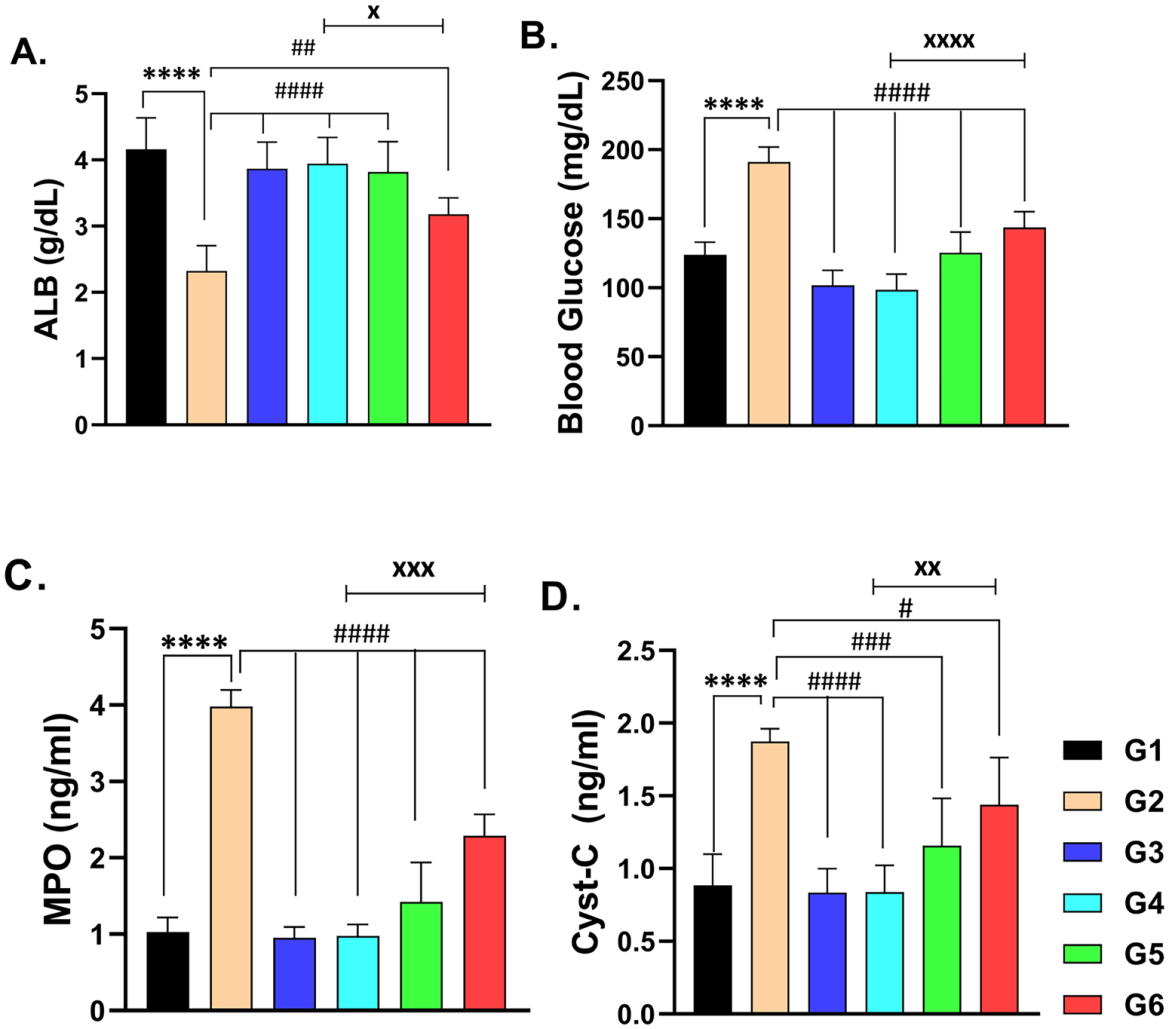
The effects of administration of cyclosporine, sitagliptin, and hesperidin on the serum levels of albumin (ALB) (**A**), blood glucose (**B**), myeloperoxidase (MPO) (**C**) and cystatin-C (Cyst-C) (**D**). data represented as mean ± SEM (n = 6). (G1 = Control, G2 = CsA-treated rats, G3 = Sitagliptin, G4 = Sitagliptin + CsA, G5 = Hesperidin, G6 = Hesperidin + CsA). ^****^ (*p* < 0.0001) versus G1 rats, ^#^ (*p* < 0.05), ^##^ (*p* < 0.01), ^###^ (*p* < 0.001) & ^####^ (*p* < 0.0001) versus G2 rats, ^xx^ (*p* < 0.01) & ^xxxx^ (*p* < 0.0001) versus G6 rats.

For serum albumin, cyclosporine A administration to rats substantially decreased its levels in comparison to control rats (Fig. 2A, *p* < 0.0001). The coadministration of sitagliptin with CsA gave rise to a significant preservation of albumin levels compared to those of CsA-treated rats (Fig. 2A, *p* < 0.0001) and to those that received a combination of CsA and hesperidin. As illustrated in Fig. 2, CsA treatment showed a deleterious impact on the levels of glucose, myeloperoxidase (MPO), and cystatin-C (CYS-C), as they were elevated significantly (Fig. 2B–D, respectively, *p* < 0.0001). Treatment with sitagliptin or hesperidin attenuated the elevation of the aforementioned parameters considerably in reference to CsA-treated animals (*p* < 0.0001). Furthermore, treatment with sitagliptin or hesperidin individually showed no significant changes in the levels of the biomarkers compared to the control rats. Such results explain the reno-protective activities of sitagliptin or hesperidin against CsA-induced damage to kidney cells. For the above-mentioned serum markers, sitagliptin administration with CsA produced a significant effect compared to hesperidin administration with CsA (Fig. 2).

### Kidney MDA and GSH

**Figure 3:**
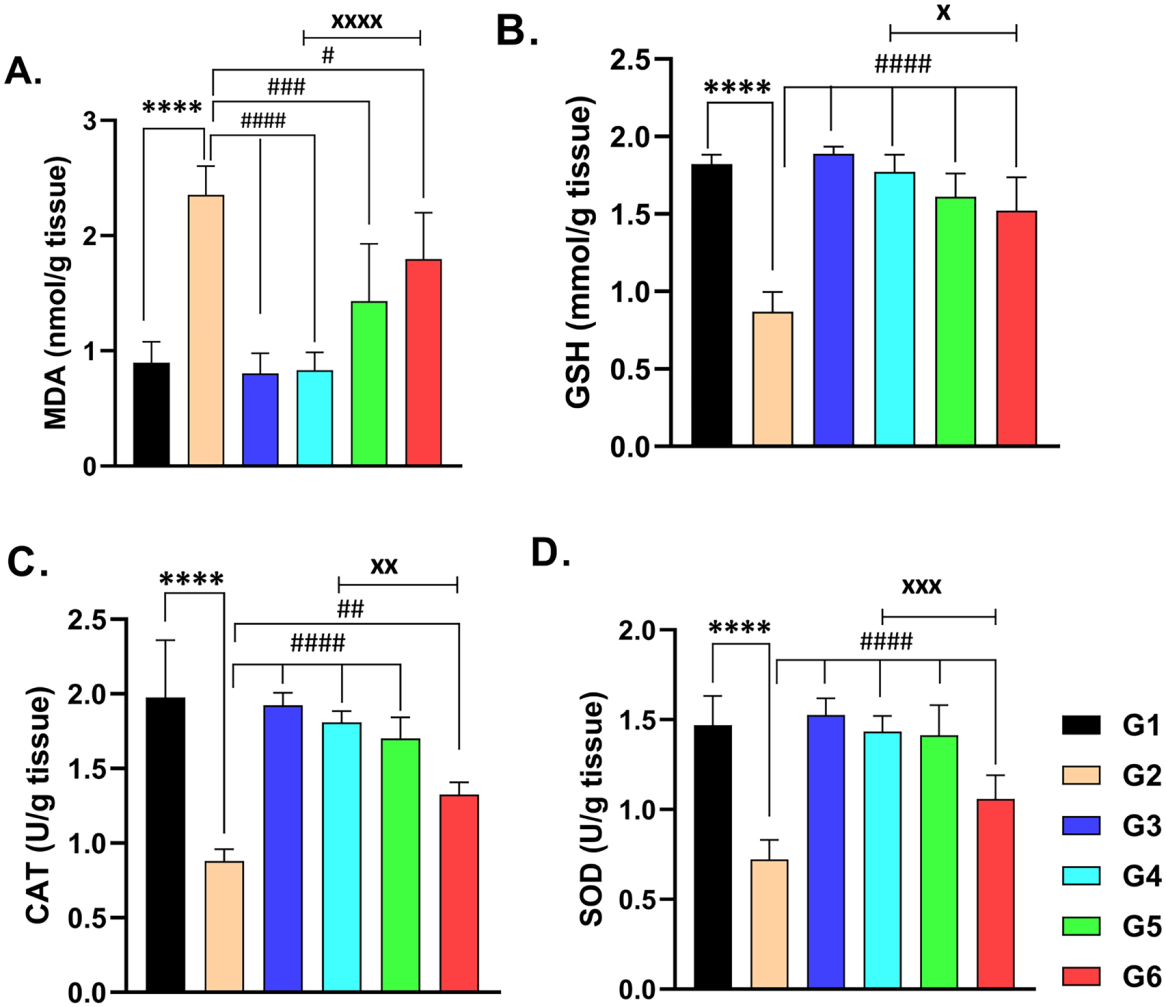
The renal tissue levels of MDA (**A**) and GSH (**B**) and renal tissue activities of CAT (**C**) and SOD (**D**) on administration of CsA, Sitagliptin, and hesperidin. Data represented as mean ± SEM (n = 6). MDA = Malondialdehyde, GSH = reduced glutathione, CAT = Catalase, SOD = Superoxide dismutase & CsA = Cyclosporine A. (G1 = Control, G2 = CsA-treated rats, G3 = Sitagliptin, G4 = Sitagliptin + CsA, G5 = Hesperidin, G6 = Hesperidin + CsA). ^****^ (*p* < 0.0001) significant difference from normal control rats, ^#^(*p* < 0.05), ^##^(*p* < 0.01), ^###^(*p* < 0.001) & ^####^ (*p* < 0.0001) significant difference from CsA-treated rats and ^x^ (*p* < 0.05), ^xx^ (*p* < 0.01), ^xxx^ (*p* < 0.001) & ^xxxx^ (*p* < 0.0001) significant difference from (Hesperidin + CsA)-treated rats.

The renal tissue of rats that received CsA showed a significant elevation of the biochemical marker of lipid peroxidation (MDA) compared to normal control rats (*p* < 0.0001) (Fig. 3A,B). Coadministration of sitagliptin with CsA resulted in a significant attenuation of CsA-induced MDA rise (*p* < 0.0001) as compared to CsA-treated rats. The renal tissue glutathione (GSH) has been reduced upon CsA treatment, indicating its depletion in comparison to control animals (*p* < 0.0001). As noted in Fig. 3B, the use of sitagliptin or hesperidin with CsA produced the preservation of kidney GSH, maintaining an antioxidant defense mechanism (*p* < 0.0001). The use of sitagliptin or hesperidin alone had no significant impact on the MDA and GSH levels, which were near normal with these medications. It is noteworthy to mention that sitagliptin has a greater effect compared to the use of hesperidin against CsA-induced oxidative insult in the kidney (Fig. 3A,B, *p* < 0.05).

### Kidney CAT activity

Measuring the CAT activity revealed the considerable defect produced using CsA in the kidney tissues, comparing this to normal kidneys in rats that received the vehicle (Fig. 3C, *p* < 0.0001). Interestingly, administering sitagliptin or hesperidin concomitantly with CsA preserved renal CAT activity (Fig. 3C, *p* < 0.0001). The use of sitagliptin or hesperidin alone produced no significant effect on the CAT activity in the renal tissue. Comparing sitagliptin with CsA to hesperidin with CsA brought out the ability of sitagliptin to have a higher beneficial effect (*p* < 0.05).

### Kidney SOD activity

When compared to control rats, CsA-treated animals showed a lowered SOD activity in the renal tissue (Fig. 3D, *p* < 0.0001). SOD activity was preserved near normal by using sitagliptin or hesperidin with CsA when compared to rats with CsA-induced nephrotoxicity (*p* < 0.0001). As noticed in Fig. 3D, the use of sitagliptin or hesperidin resulted in insignificant changes in SOD activity compared to normal rats. Additionally, sitagliptin preserved SOD activity more than hesperidin did when given with CsA.

### Renal tissue TNF-α

**Figure 4:**
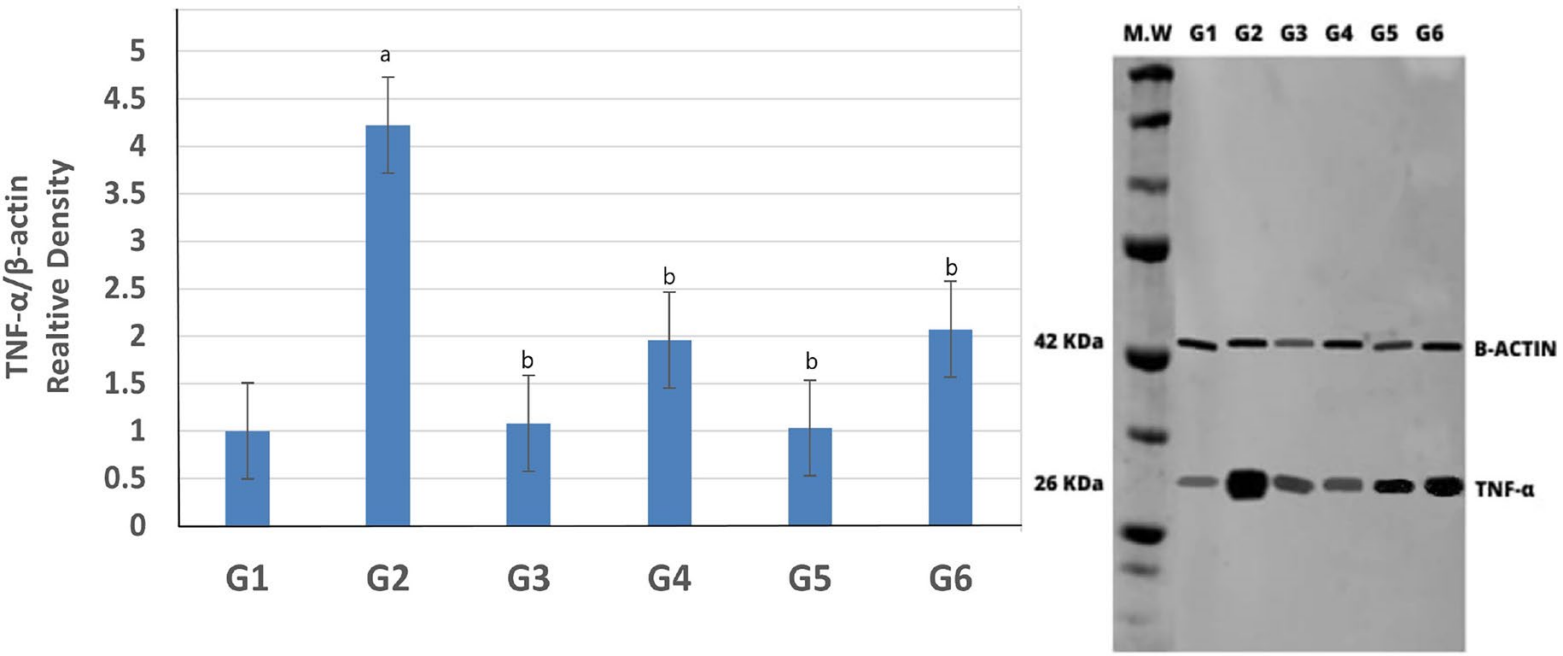
Effect of sitagliptin and hesperidin on expression of TNF-α in CsA treated rats. Immunoblotting for the target molecule of TNF-α showing the effects of sitagliptin and hesperidin in CsA-treated rats. Bar graphs show the expression levels of TNF-α. Β-actin was used as the internal control. (G1 = Control, G2 = CsA-treated rats, G3 = Sitagliptin, G4 = Sitagliptin + CsA, G5 = Hesperidin, G6 = Hesperidin + CsA). ^a^(*p* < 0.001) compared to the control group, and ^b^(p < 0.05) compared to the CsA group.

To further substantiate the protective effects associated with sitagliptin or hesperidin against CSA-induced nephrotoxicity in rats, protein expression of TNF-α was assessed. Notably, CsA significantly induced the expression of TNF-α by approximately 200% relative to the control values. The expression of TNF-α in the kidney tissue was higher in rats receiving CsA compared to control animals. The administration of sitagliptin or hesperidin with CsA resulted in a significant reduction in TNF-α expression in the kidney tissues when compared to CsA-treated rats. When administered alone, sitagliptin or hesperidin produced no significant changes compared to control rats. It is to be mentioned that sitagliptin treatment was slightly superior to hesperidin administration when given with CsA (Fig. 4).

### Histopathological examination

**Figure 5:**
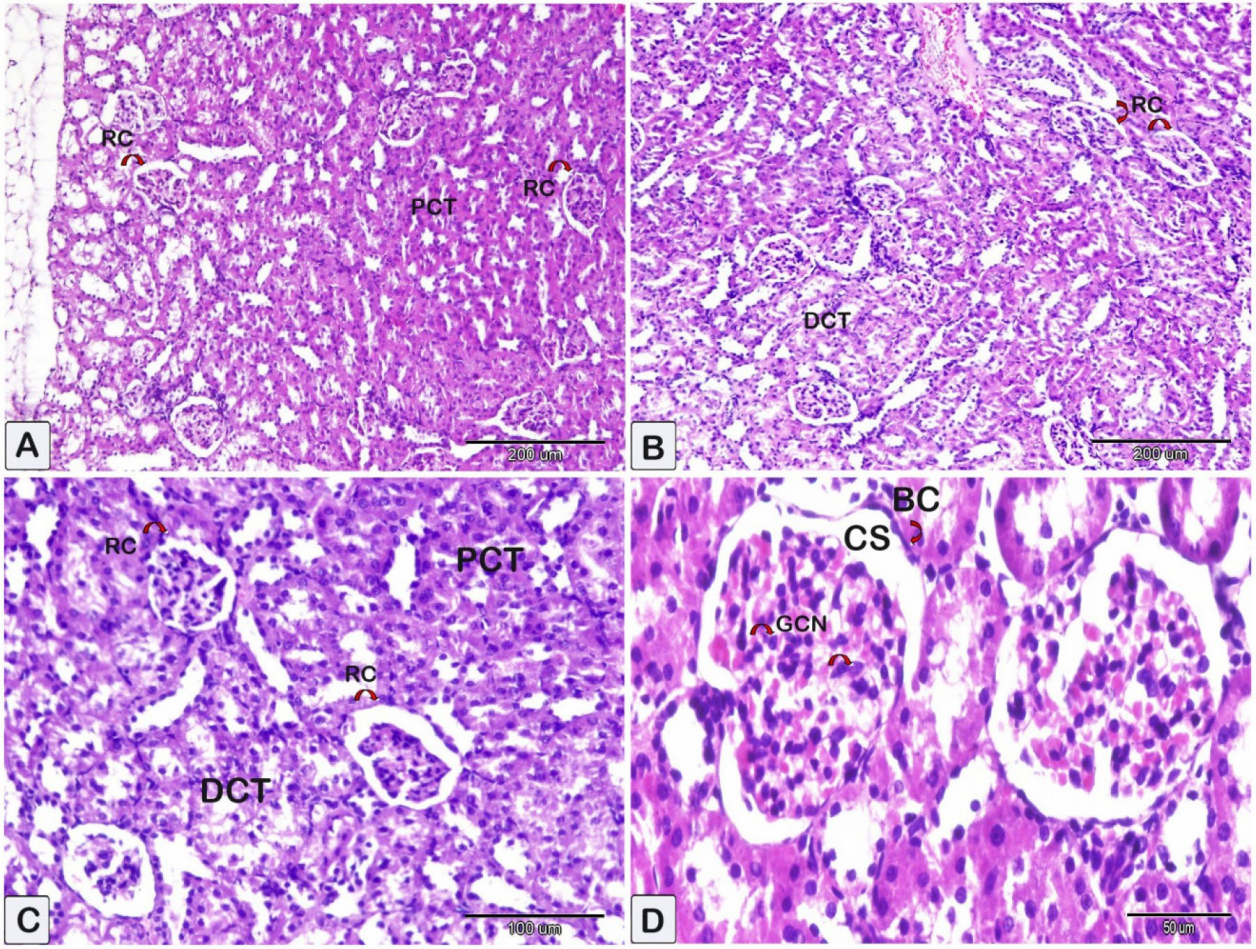
Representative photomicrographs of paraffin sections of kidney tissue obtained from the control group. These sections were stained with hematoxylin and eosin (HE). Microscopic examinations of the kidney tissues from control rats that received the vehicle revealed that the renal glomeruli and proximal and distal convoluted tubules had normal histological appearances. (**A**–**D**): The renal corpuscle (RC), consisting of Bowman’s capsules (BC), demonstrates well-defined and consistent structural features. A typical epithelial cell with proximal (PCT) and distal convoluted tubules (DCT) is observed. The abundance of glomerular cell nuclei (GCN, shown by curved arrows) is notable. (CS) referred to capsular space.

**Figure 6:**
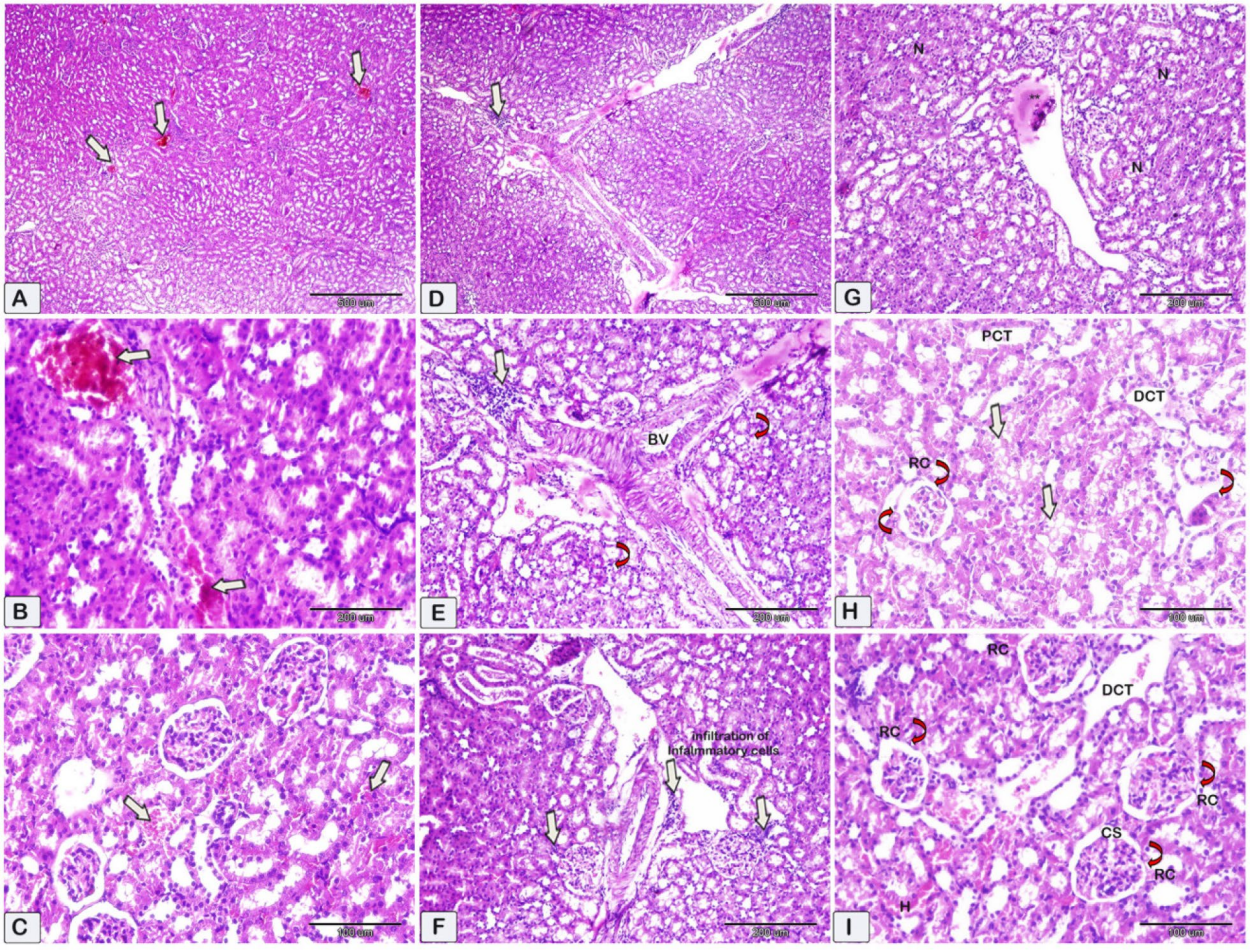
Representative photomicrographs of paraffin sections of kidney tissue obtained from the nephrotoxicity group (CsA group). These sections were stained with hematoxylin and eosin (HE) to visualize the tissue. (**A**,**B**) Vascular congestion and (**C**) Interstitial hemorrhages (shown by white arrows). The observed pathological findings include perivascular inflammatory cell infiltration, shown by the presence of white arrows surrounding blood vessels, as well as tubular degeneration accompanied by necrosis of epithelial cells, as denoted by the curved arrows. (**D**–**I**) the presence of necrosis in kidney tissue (N) is observed, namely in the proximal (PCT) and distal convoluted tubules (DCT). This necrosis is accompanied by a reduction in the size of the glomeruli within the renal corpuscle (RC, indicated by curved arrows) and a narrowing of the capsular space (CS). Additionally, there is evidence of interstitial hemorrhage (H) in the shown image in Figure (I). Macrophage Ginat cells were observed in Figure H (red curved arrow).

**Figure 7:**
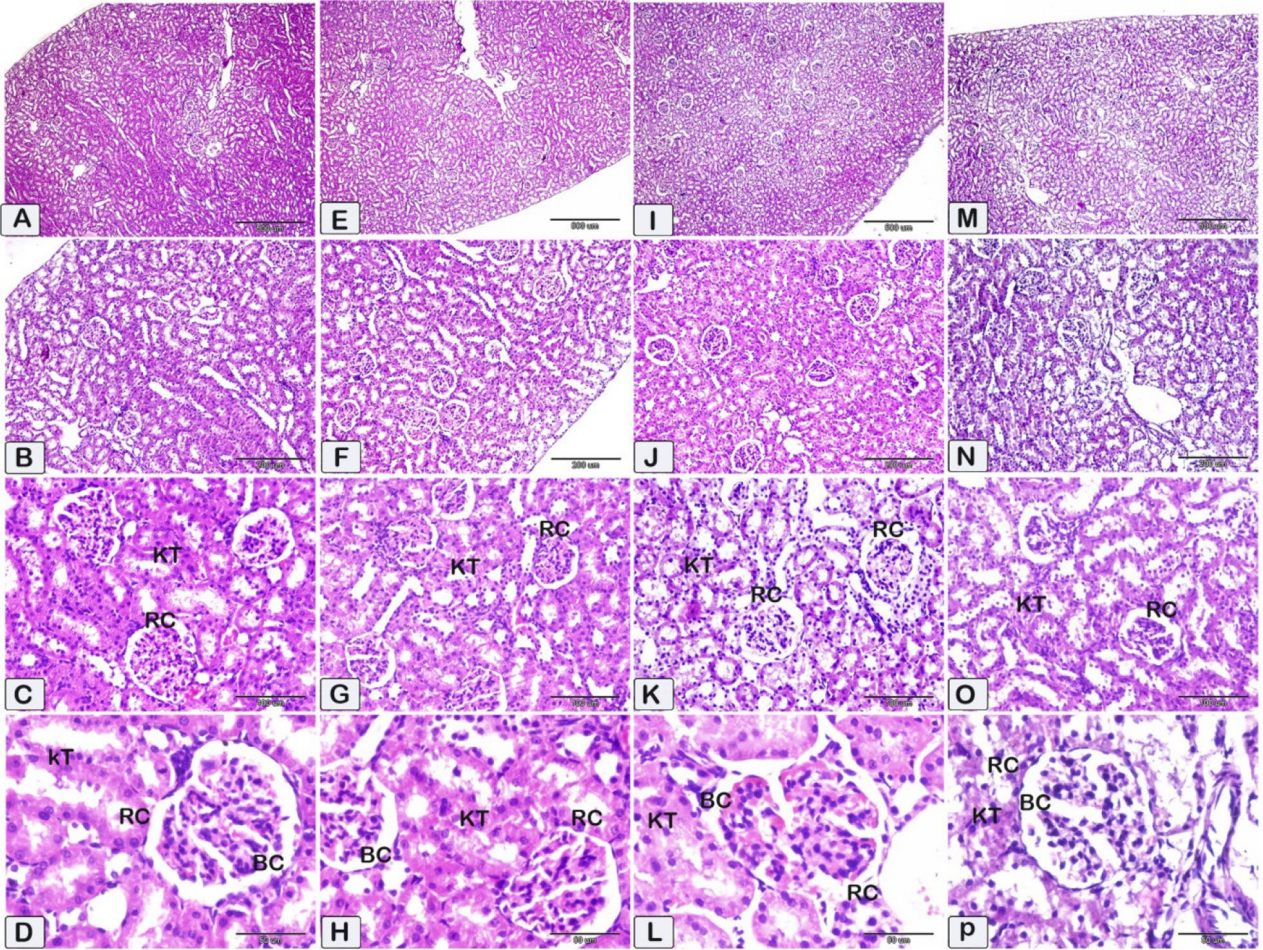
Displayed photomicrographs of paraffin sections of kidney tissue obtained from the treated groups and stained using hematoxylin and eosin (HE). (**A**–**D**) Figures at low and high magnifications of the kidney cortex of experimental group 3. (**E**–**H**) Figures at low and high magnifications of kidney cortex of experimental group 4. (**I**–**L**) Figures at low and high magnifications of the kidney cortex of experimental group 5. (**M**–**P**) Figures at low and high magnifications of the kidney cortex of experimental group 6 illustrate the typical histological features of kidney tubules and renal corpuscles within the cortex.

The photomicrographs of kidney sections from the control group of rats exhibited normal glomeruli with an intact Bowman’s capsule, as well as normal proximal convoluted tubules and distal convoluted tubules in the renal cortex (Fig. 5). The renal cortex sections of rats that received CsA exhibited observable manifestations such as glomerular shrinkage, a decrease in Bowmen’s space, and renal corpuscles. The lining epithelium of several tubules exhibited disruption, resulting in dilation and large necrotic regions. The renal tubules displayed dilation, accompanied by the presence of interstitial inflammatory cells surrounding blood vessels, vascular congestions, and interstitial hemorrhage and the detection of macrophage giant cells (Fig. 6). These findings were greatly obvious when compared to control animals (Fig. 6 vs. Fig. 5). Examination of the kidney sections from other groups revealed that sitagliptin or hesperidin administration was not associated with significant pathological findings as compared to CsA-treated rats. When sitagliptin or hesperidin were given with CsA, they attenuated the development of pathological kidney damage compared to the nephrotoxicity group, and this was more obvious regarding sitagliptin treatment with CsA (Fig. 7).

**Figure 8:**
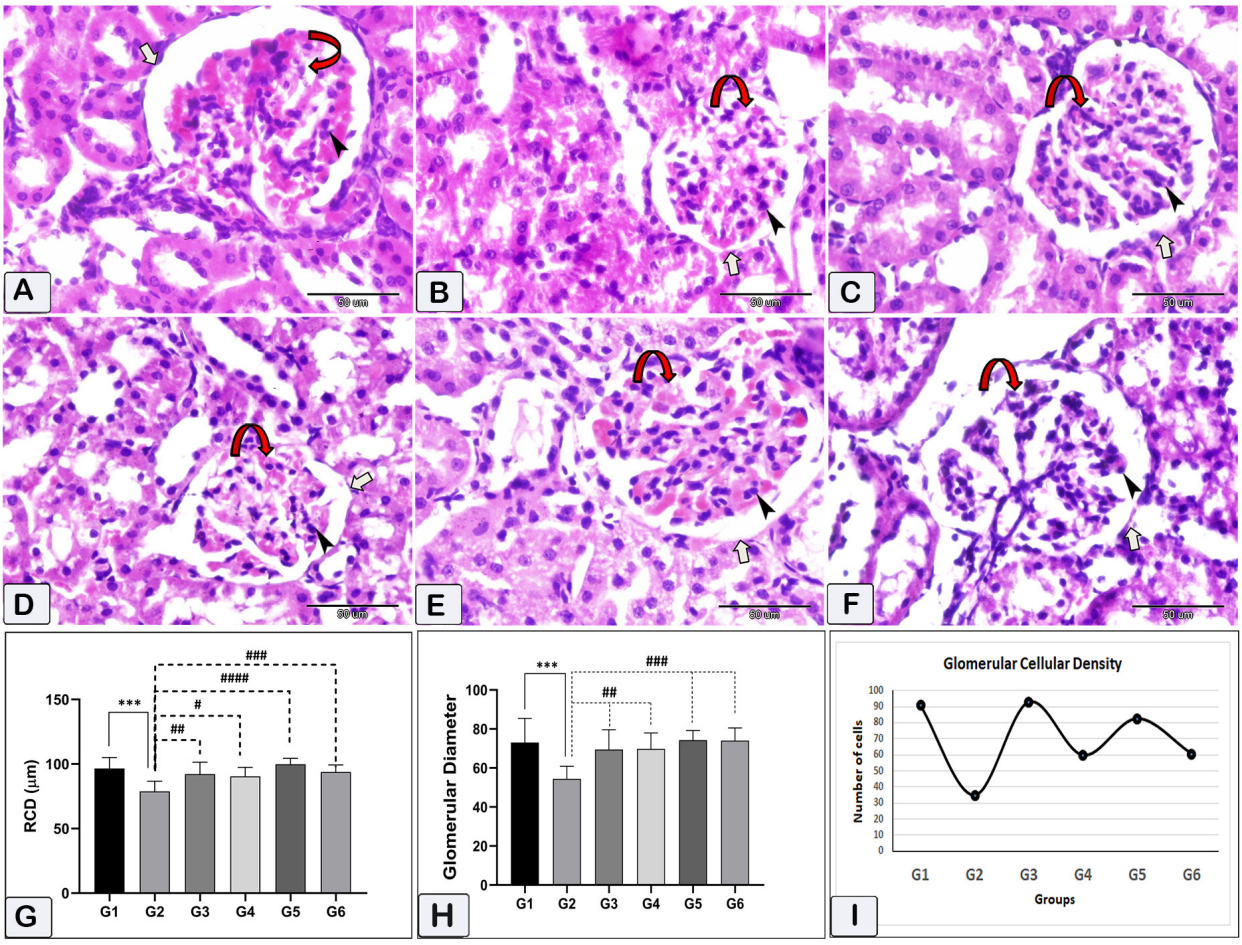
Kidney tissue photomicrographs stained with hematoxylin and eosin (HE). Renal corpuscle diameter, glomerular diameter, and glomerular cell density within the glomeruli were visualized. And estimated. This was achieved by quantifying the number of cell nuclei to assess these parameters. (**A**) G1, (**B**): G2, (**C**) G3, (**D**) G4. (**E**) G5. & (**F**) G6. The curved arrows indicate the location of the glomeruli, black arrowheads indicate the nuclei within the glomeruli, and white arrows indicate the renal corpuscle. G: the renal corpuscle diameter (RCD). (**H**) the glomerular diameter. (**I**) the glomerular cell density. *** (*p* < 0.001) Significant difference from control rats. # (*p* < 0.05), ## (*p* < 0.01), ### (*p* < 0.001) & #### (*p* < 0.0001) Significant difference from CsA group. G1 = Control, G2 = CsA-treated rats, G3 = Sitagliptin, G4 = Sitagliptin + CsA, G5 = Hesperidin, G6 = Hesperidin + CsA.

**Figure 9:**
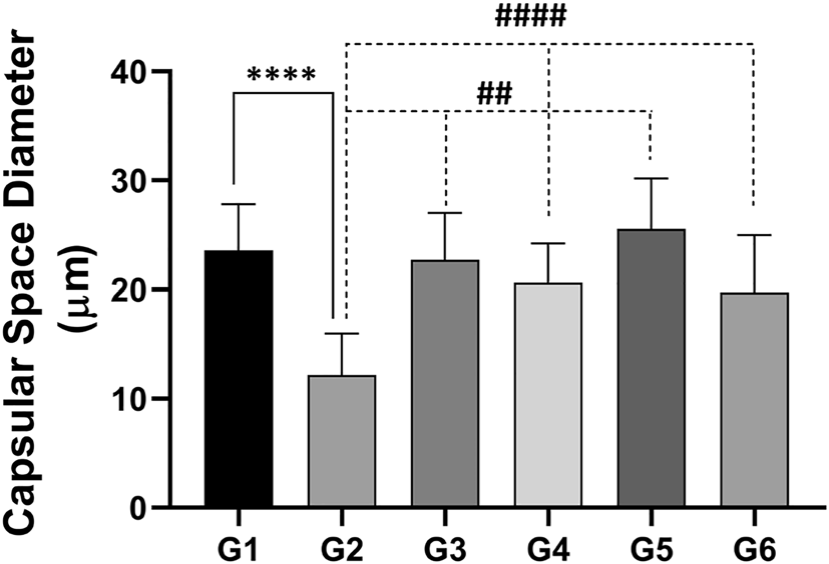
The effect of CsA administration on the capsular space diameter and the potential modification obtained by administering sitagliptin or hesperidin either alone or with CsA. Data represented as mean ± SEM. *** (*p* < 0.001) Significant difference from control rats. ## (*p* < 0.01), #### (*p* < 0.0001) Significant difference from CsA group. G1 = Control, G2 = CsA-treated rats, G3 = Sitagliptin, G4 = Sitagliptin + CsA, G5 = Hesperidin, G6 = Hesperidin + CsA.

The histological measurements uncovered the shrinkage of the renal corpuscles in the rats that received CsA when compared to control rats. The glomerular diameter and capsular space were markedly lower in the nephrotoxicity group than in healthy rats. Treatment with sitagliptin, hesperidin alone, or coadministered to CsA resulted in near-to-normal glomerular diameter and capsular space diameter (Figs. 8 and 9).

**Figure 10:**
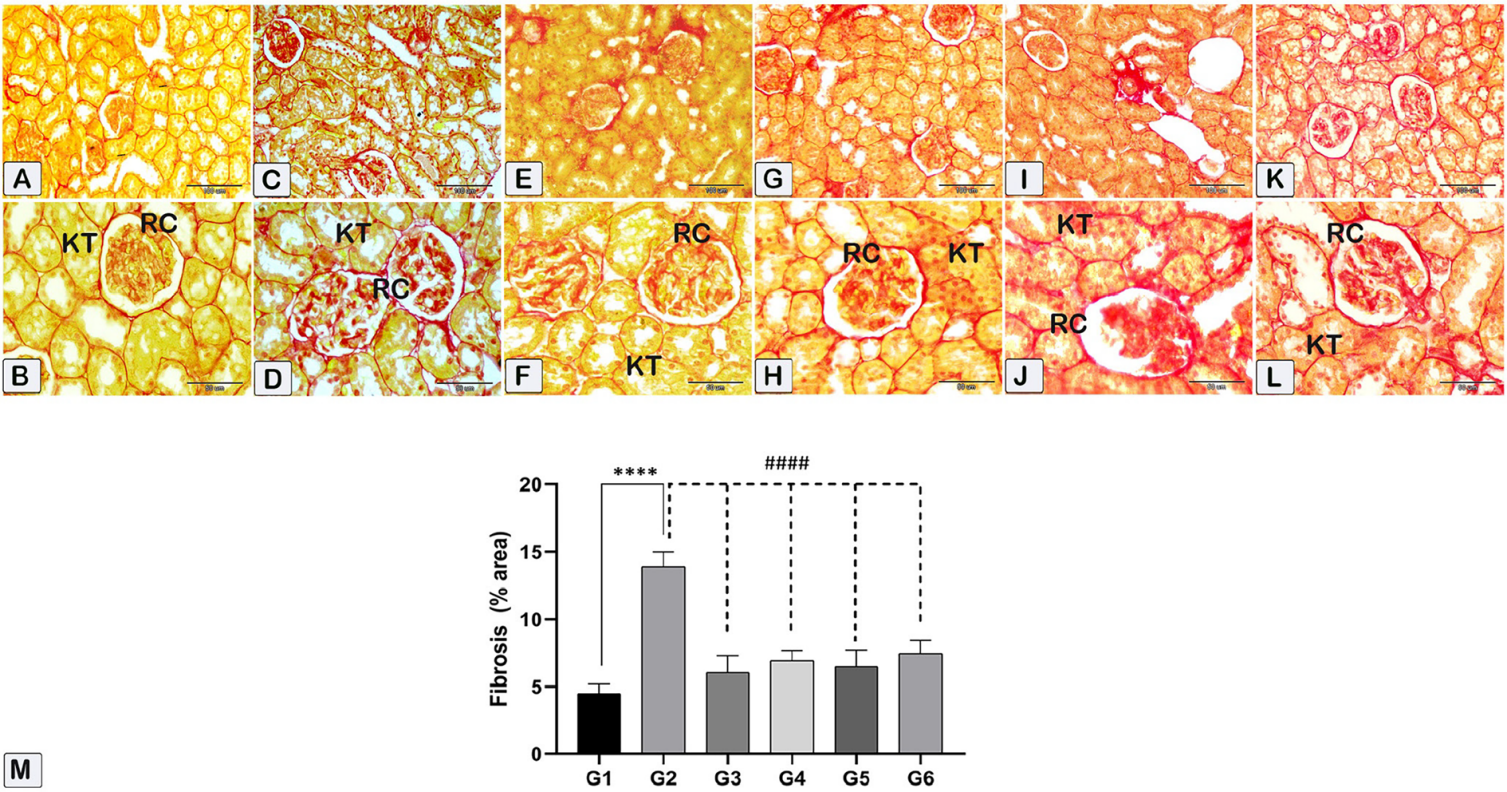
The kidney sections were stained with Sirius Red to measure fibrosis. Red collagenous fibers surround kidney tubules (KT), basement membrane, and renal corpuscles (RC). Histological (**A**–**L**) and morphometric assessments of all experimental groups (**M**) showed that the control group had fewer collagenous fibers than the nephrotoxicity group. However, the fibrosis percentage increased in the nephrotoxicity group and decreased in the treated groups. (**A**,**B**) In the control group (G1), kidney tubules and renal corpuscles stained less with collagen fibers than in the toxicity CsA group. (**C**,**D**) In the nephrotoxicity group (G2), kidney tubules and renal corpuscles stained more with collagen fibers than in the control group. In G3 (**E**,**F**), G4 (**G**,**H**), G5 (**I**,**J**), and G6 (**K**,**L**), a notable decrease in collagen fiber staining was detected in the kidney tubules and renal corpuscles as compared to the nephrotoxicity group. Nevertheless, the observed quantity seemed to be relatively comparable to that observed in the control group. (**M**) Mean area of morphometric analysis of collagenous fibers % of kidney sections in different groups. **** (*p* < 0.0001) Significant difference from control rats. #### (*p* < 0.0001) Significant difference from CsA group. G1 = Control, G2 = CsA-treated rats, G3 = Sitagliptin, G4 = Sitagliptin + CsA, G5 = Hesperidin, G6 = Hesperidin + CsA.

**Figure 11:**
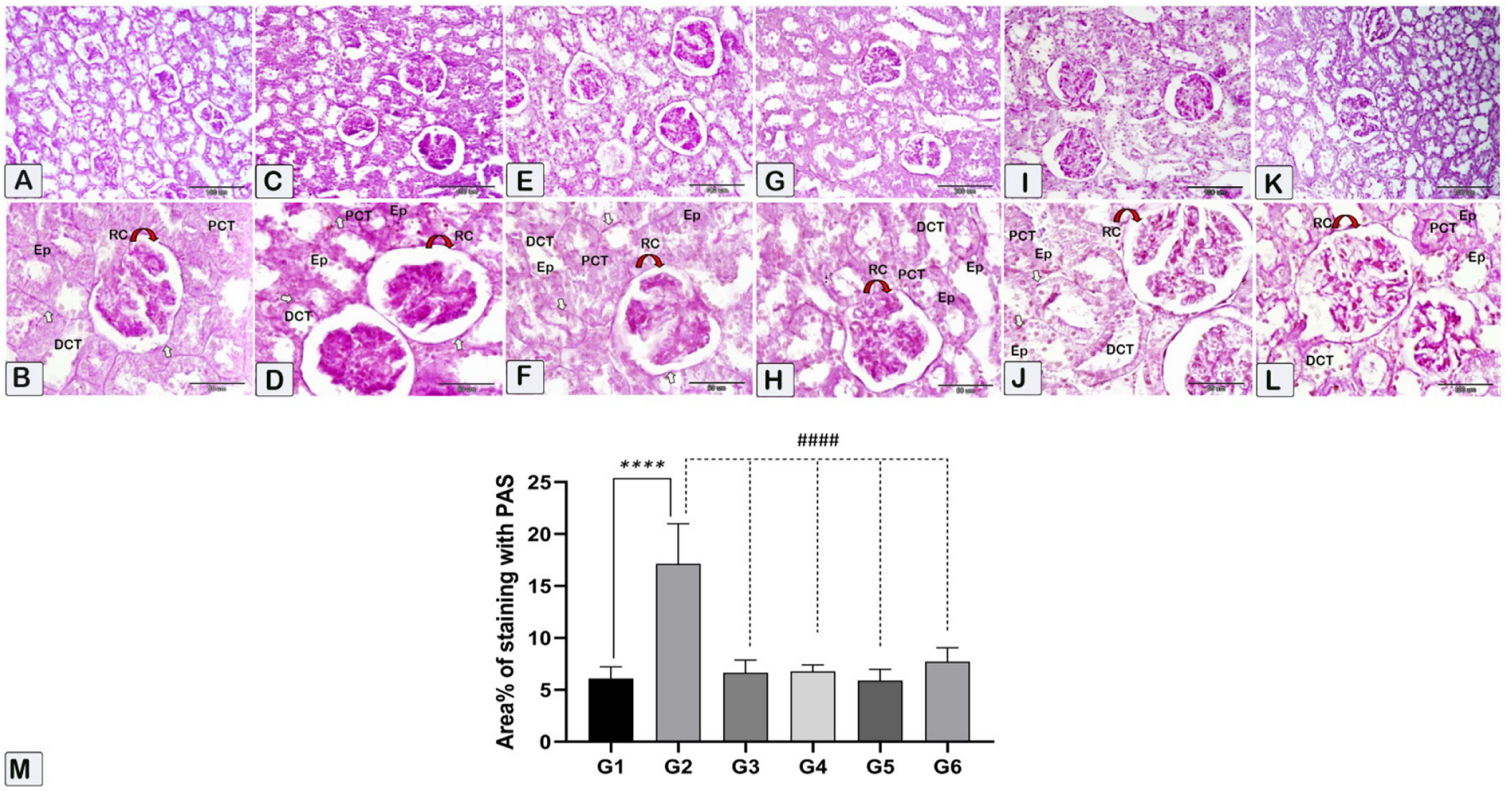
Photographs of paraffin-embedded kidney tissue from the experimental groups are presented. PAS stain was used to color the tissue sections. Brush profits, renal tubule basement membranes, Bowman’s capsule, and glomerular tufts of capillaries all show a rich purple color due to the high polysaccharide content of the kidney cortex. (**A**,**B**) In the control group (G1), kidney tubules and renal corpuscles stained less than in the toxicity group. (**C**,**D**) In the nephrotoxicity group (G2), kidney tubules and renal corpuscles stained more with PAS stain than in the control group. In G3 (**E**,**F**), G4 (**G**,**H**), G5 (**I**,**J**), and G6 (**K**,**L**), a notable decrease in staining intensity percentage was detected in the kidney tubules and renal corpuscles as compared to the nephrotoxicity group. (**M**) Mean area of morphometric analysis of staining intensity % of kidney sections in different groups. G1 = Control, G2 = CsA-treated rats, G3 = Sitagliptin, G4 = Sitagliptin + CsA, G5 = Hesperidin, G6 = Hesperidin + CsA.

The present study involved the determination of the collagenous fibers using histomorphometric analysis as well as the quantification of fibrosis percentage in kidney sections. This analysis is conducted using the Sirius Red stain (Fig. 10) and the Periodic Acid-Schiff (PAS) stain (Fig. 11) to assess the percentage reaction in the kidney sections. The data analysis is represented as means ± standard error (SE) and is visually depicted in Figs. 8M and 9 M. The fibrosis and PAS stain intensity were increased in the CsA group, followed by a significant decrease in various treatment groups, and nearly returned to the control level. Furthermore, it was noticed that sitagliptin administration with CsA showed improved histological outcomes when compared to hesperidin treatment.

### Immunohistochemical examination

#### Renal Nrf2

**Figure 12:**
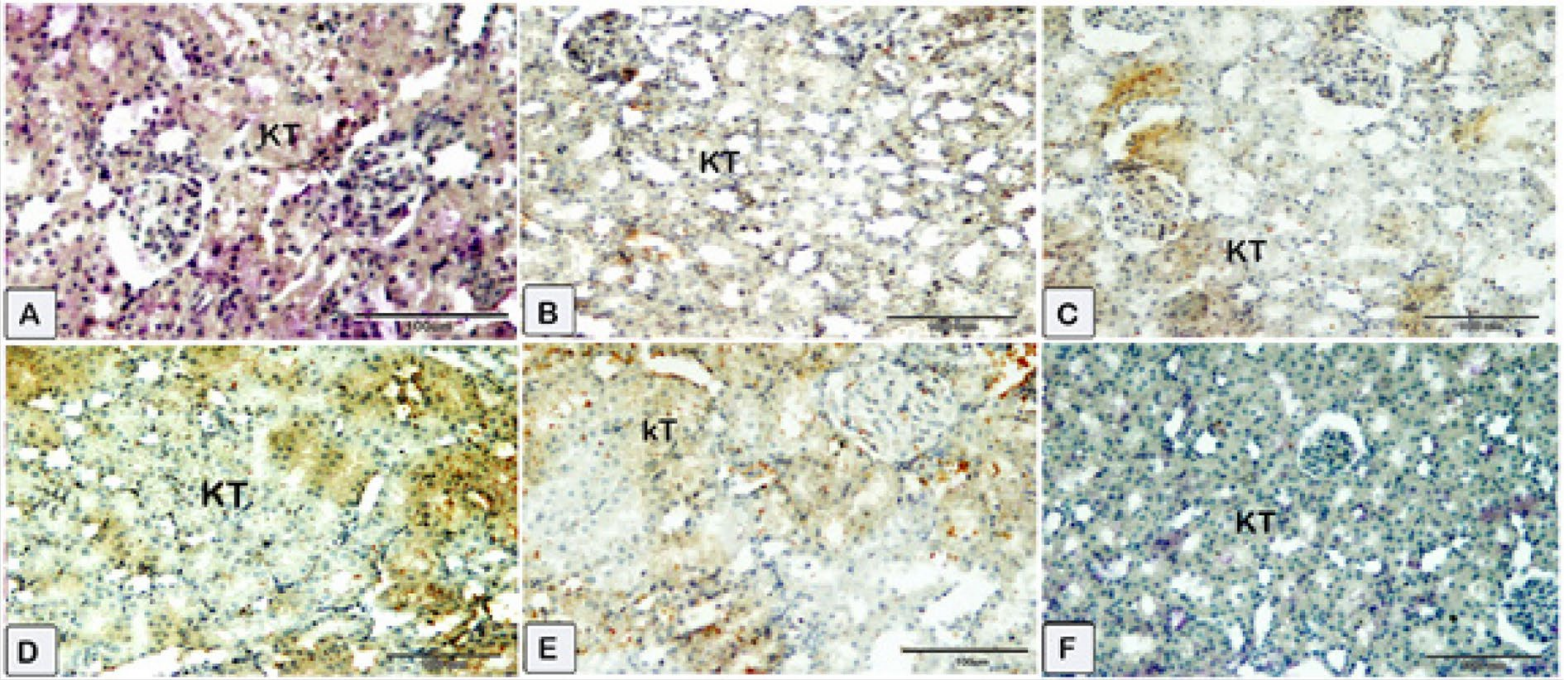
Representative photomicrographs of immunohistochemistry displaying the expression of Nrf2 protein through the application of immunohistochemistry. (**A**) Sections of the kidney from the control showed average Nrf2 expression (**B**) Sections of the CsA group showed that renal tubular cells’ cytoplasm and nucleus exhibit low levels of Nrf2 expression. (**C**) Sections of sitagliptin alone, and renal tissues showed near control degree of Nrf2 expression. (**D**) Sections of the sitagliptin/CsA group showed an average level of Nrf2 expression. (**E**) Sections of hesperidin alone revealed a similar response as sitagliptin. (**F**) Sections of the hesperidin/CsA group showed that brown staining was raised in intensity. (**A**) Control group (G1) (**B**) CsA group (G2), (**C**) Sitagliptin group (G3), (**D**) Sitagliptin + CsA (G4), (**E**) Hesperidin group (G5), (**F**) Hesperidin + CsA (G6).

Immunostaining of renal tissue demonstrated observable levels of Nrf2 expression in the control rats (Fig. 12A). Sections of the CsA group (Fig. 12B) show renal tubular cells’ cytoplasm and nuclei exhibit faint immunoexpression of Nrf2. Treatment with sitagliptin or hesperidin revealed near-to-control stained immune-reactive cells of renal tissues (Fig. 12C,E). Coadministration of sitagliptin to CsA showed enhanced Nrf2 expression as compared to CsA-treated rats (Fig. 12D). Furthermore, the presence of hesperidin along with CsA gave rise to obviously increased protein expression of Nrf2 (Fig. 12F). Additionally, it was detected that sitagliptin treatment with CsA produced a superior improvement in the protein expression of Nrf2 compared to hesperidin treatment with CsA.

#### Renal Bax

**Figure 13:**
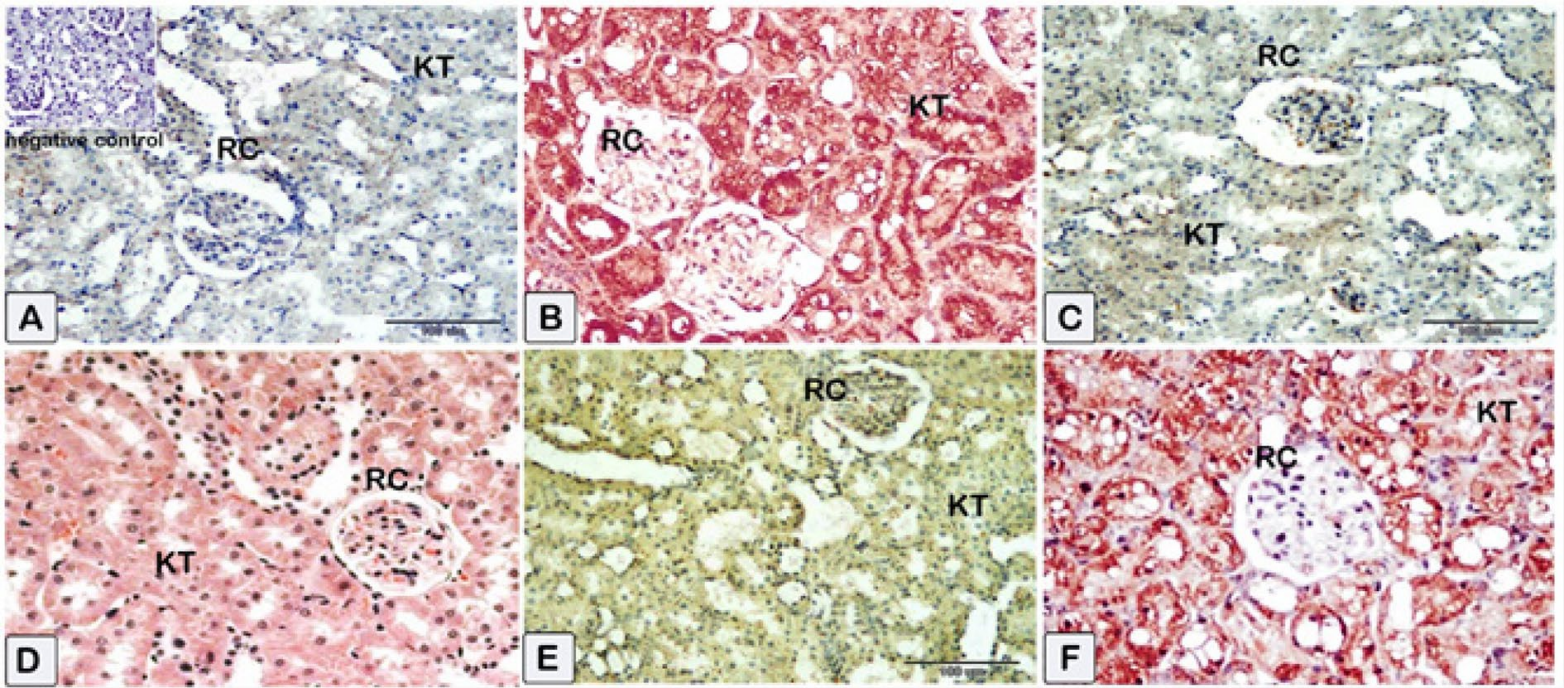
Representative photomicrographs of immunohistochemistry were done to illustrate the mean immunoexpression levels of Bcl-2-associated × protein (Bax). Results of cytoplasmic Bax positivity in each experimental group (X40) as determined. A substantial positive cytoplasmic Bax reaction was observed in glomerular and tubular cells of the nephrotoxicity group (**B**), in contrast to the negative reaction observed in the control group (**A**). The cytoplasmic Bax reaction in glomerular and tubular cells was reduced in rats treated with (**C**–**F**). (**A**) Control group (G1) (**B**) CsA group (G2), (**C**) Sitagliptin group (G3), (**D**) Sitagliptin + CsA (G4), (**E**) Hesperidin group (G5), (**F**) Hesperidin + CsA (G6).

The expression of renal Bax was evaluated in the kidneys of the different groups. Glomerular and tubular cells from the nephrotoxicity group displayed an impressive positive cytoplasmic Bax reaction (Fig. 13B) compared to the negative reaction observed in the control group (Fig. 13A). Kidney sections of rats received sitagliptin or hesperidin uncovered renal tissues showed few immune-reactive cells with low Bax expression (Fig. 13C,E). The sitagliptin/CsA group’s kidney sections had extremely low Bax immunoreactivity (Fig. 13D). The kidney tissues of the hesperidin/CsA group also showed decreased Bax expression and decreased brown staining intensity (Fig. 13F).

#### Renal NF-κB

**Figure 14:**
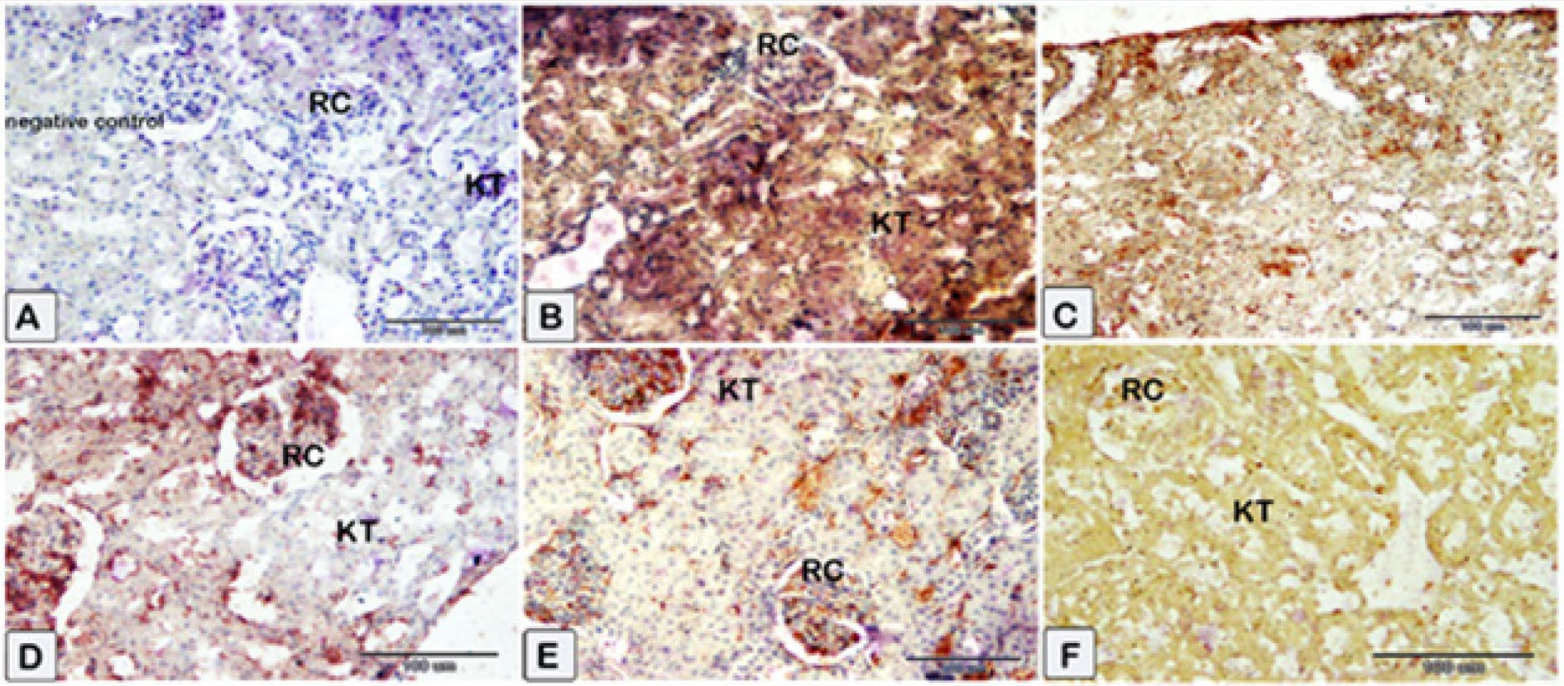
Representative photomicrographs of paraffin sections of kidney tissue acquired from the experimental group, displaying the expression of NF-κB protein through the application of immunohistochemistry. Group-specific variations in renal NF-κB protein expression were observed. Reduced levels of the NF-κB protein are observed control group. The expression of the NF-κB protein was higher in the nephrotoxicity group than in the control group. Following this, the treated group’s expression levels of NF-κB decreased. (**A**) Control group (G1) (**B**) CsA group (G2), (**C**) Sitagliptin group (G3), (**D**) Sitagliptin + CsA (G4), (**E**) Hesperidin group (G5), (**F**) Hesperidin + CsA (G6).

In the control group, a decrease in the levels of the NF-κB protein is detected (Fig. 14A). The levels of NF-κB protein expression were found to be significantly elevated in the CsA group compared to the control group (Fig. 14B). Subsequently, the expression levels of the treated group exhibited a reduction in the protein expressions of NF-κB, as indicated by a low level of staining intensity and reaction in sections from rats received sitagliptin or hesperidin individually or with CsA (Fig. 14C–F). It was noticed that the immune reaction for NF-κB was lower in the kidney sections of rats that received sitagliptin with CsA than in the kidney sections of rats that received hesperidin with CsA.

## Discussion

The most serious limiting side effect of cyclosporine A (CsA) is nephrotoxicity^48^, which can be manifested as either acute reversible kidney injury or chronic fibrotic renal dysfunction^49^. It involves lesions such as tubular atrophy and interstitial fibrosis^50^. Several interventions have been introduced to halt the pathogenesis of this dangerous complication during therapy with cyclosporine in patients with autoimmune diseases and organ transplantation^3^. Cyclosporine induces oxidative stress, which acts in various ways to cause tissue injury. Inflammatory cell infiltration into the kidney is a common finding, and this is initially associated with augmented expression and release of inflammatory cytokines and chemokines in the kidney^51^. In this work, we sought to determine if sitagliptin and hesperidin may shield rat kidneys from the damaging effects of cyclosporine. This was an attempt to protect kidney structure and function when using cyclosporine to treat an autoimmune illness or during organ transplantation. Urine and creatinine levels in the serum were significantly elevated in rats given cyclosporine. This was in line with numerous studies that triggered renal damage in animal models using cyclosporine^19,52,53^.

Cyclosporine caused a significant elevation in serum Cr, BUN, and urinary protein with a significant decrease in serum albumin^54,55^. Also, cyclosporine injection into rats produced elevations in lipids, and glucose while decreasing albumin and protein levels^56,57^. The main pathogenesis of CsA-induced hyperglycemia is caused by the direct toxicity of CsA on pancreatic beta cells, which leads to a decrease in insulin production^58,59^; while insulin resistance plays a minor role in this phenomenon^60^. Our results are consistent with previously mentioned studies, and it was concluded that inhibition of calcineurin might play an important role in the inhibition of the synthesis and release of insulin with hyperglycemia and manifested diabetes^61^.

CsA-treated animals showed a spike in MPO activity, indicating renal invasion with inflammatory leukocytes and parenchymal structural derangements^62^. This enzyme is responsible for generating hypochlorous acid during the neutrophil burst reaction, which consequently increases free radical generation and subsequent tissue damage. An increase in the levels of MPO is concomitant with elevated tissue levels of TNF-α, thereby indicating the role of inflammatory cells in organ damage caused by CsA^8,63^. Daily injections of CsA resulted in significant deterioration of the kidney with elevated serum cystatin C^64^. Our results showed that CsA treatment significantly raised serum levels of Cyst-C and MPO, indicating deteriorated renal function compared with the control rats.

Administration of cyclosporine-A to rats resulted in a dramatic alteration in redox homeostasis, as evidenced by a decrease in tissue antioxidants, GSH, and SOD, and an increase in the levels of the lipid peroxidation marker MDA. These effects of CsA have been attributed to the disruption of mitochondrial oxidative phosphorylation with ROS generation^2,8^. CsA increases ROS production in several cellular models, decreasing antioxidant levels and inducing lipid peroxidation^11,65,66^. Also, other studies found that the oxidative parameters in the renal tissues and sera of animals treated with cyclosporine were significantly increased^67,68^.

The decline in renal tissue SOD activity after CsA administration was also reported in some other studies^69^, and treatment with the SOD mimetic tempol was able to prevent CsA-induced renal dysfunction^70^. In addition, there was decreased serum SOD activity in rats treated with CsA^71^. Also, CsA administration to experimental animals resulted in elevated MDA in kidney tissues with lowered CAT activity^48^. Furthermore, CsA produced not only elevated tissue levels of MDA and nitric oxide but also a significant reduction in the tissue levels of several antioxidant enzymes, including CAT, SOD, and GPx. Likewise, CsA led to the accumulation of ROS in the kidney, which directly contributes to renal damage caused by CsA. Growing data suggests that the main mechanism behind CsA-induced nephrotoxicity is apoptosis^1,72,73^.

The glomerular mesangial cells in the kidneys are responsible for the formation of TNF-α, which reduces glomerular blood flow and glomerular filtration rate. Glomerular fibrin deposition because of a decreased glomerular filtration rate results in cellular infiltration and vasoconstriction^74,75^. As it is evaluated by western blotting, TNF-α expression in the kidney tissues was greatly increased due to CsA administration. In agreement with previous studies, the same finding was determined while investigating the renal effect of CsA^76^. CsA‑induced TNF‑α higher expression could contribute to the induction of oxidative stress‑related apoptosis and renal damage^77,78^. In addition, it is reported that TNF‑α mediates caspase 3/7 activation^79^. CsA‑induced renal inflammatory damage is presented by increased TNF‑α and lowered adiponectin levels, as well as the aggregation of inflammatory cells with renal vascular congestion^76^.

Rats treated with cyclosporine exhibited enhanced renal inflammation, as evidenced by a significant elevation of pro-inflammatory cytokines such as MCP-1 and TNF-α^80^. Concurrently, the inflow of activated macrophages and monocytes produces excess pro-inflammatory cytokines, including MCP-1 and TNF-α; these events cause and maintain increased inflammatory reactions in various renal injury models^81^.

Kidneys from rats treated with CsA showed wide capsular space, mesangial lobulation, and shrinking hypercellular glomeruli. The tubules showed diffuse degeneration with focal tubular atrophy and tubular edema, and the stroma showed infiltration by inflammatory cells^1^. Tubulointerstitial injury is the most prominent feature of chronic CsA-induced nephropathy, and the major form of cell death is apoptosis^82^. Histologically, there was tubular degeneration, atrophy, basement membrane thickening, and inflammatory cellular infiltration with renal parenchymal changes. Together, are in harmony with the results of previous studies.

Oxidative stress can cause cellular apoptosis through mitochondrial-dependent pathways^83–85^. High protein expression of Bax with decreased Bcl-2 was revealed in the renal tissue of the CsA-treated animals^1,73^. Subsequently, this study and other studies by Shihab et al.^86^ and Huang et al.^87^ reported that CsA-induced renal cell apoptosis is associated with an increase in Bax proteins. Increased Bax expression in this study reflects the increased apoptotic death of renal cells. These findings also correlated well with studies done by Temel et al.^88^. In addition, it was shown that via the redox-sensitive Nrf2 pathway, H9c2 cells were protected against apoptosis^89^. On the contrary, there was an increase in Nrf2 mRNA levels in all the CsA-treated groups; Nrf2 is a transcription factor that controls the expression of antioxidant enzymes^90^, and it is very plausible that Nrf2 is produced to decrease the ROS damage induced by the CsA-hypoxic state ^91^. As we found, administration of CsA increased Bax levels in the kidneys; this was associated with reduced expression of Nrf2. We found that the administration of CsA to rats produced increased oxidative damage, an inflammatory response, and inflammatory cellular infiltration within renal tissue. While the expressions of TNF-α and Bax were induced, the expression of Nrf2 declined significantly. The pro-inflammatory NF-κB pathway, which plays a critical role in the pathogenesis of CsA nephrotoxicity, was examined to shed light on the molecular processes behind CsA-triggered inflammatory events^81,92,93^. In line with our findings, NF-κB protein expression was significantly upregulated following CsA treatment compared to control animals^94^.

We found that hesperidin ameliorated the elevated serum levels of urea and creatinine. Hesperidin exhibited renal protective effects in several previous experiments. Küçükler et al. (2021) found hesperidin treatment considerably alleviated chlorpyrifos-induced renal dysfunction. Also, hesperidin administration relieved the kidney damage induced by paclitaxel and sodium fluoride, lowering blood urea and serum creatinine^39,95^. Hesperidin treatment significantly decreased the blood glucose, serum insulin, and HOMA-IR values in high-fat diet-induced obese mice^96^. Additionally, oral administration of hesperidin produced a significant amelioration of plasma glucose, insulin, and HbA1c levels compared with the diabetic animals^97^. It was found that hesperidin has potential antihyperglycemic activity in streptozotocin-induced diabetic rats^98^.

Sahu et al. reported that hesperidin ameliorated neutrophil infiltration, as evidenced by a reduction of MPO levels in kidney tissues. Also, Kamel et al.^99^ suggested that hesperidin administration decreased inflammatory cell infiltration in kidney tissues of rats with cisplatin-induced nephrotoxicity. Hesperidin produced a significant reduction of renal MDA and TNF-α, while it led to a significant elevation of GSH, SOD, and CAT^45^. Hesperidin significantly reduced mRNA expression levels of NF-κB, TNF-α, and Bax, whereas it caused an increase in levels of Nrf2, HO-1, and Bcl-2 in the kidney and liver of paclitaxel-treated rats^95^. Additionally, in anti-tubercular drug-induced hepatic injury, hesperidin reduced the expressions of Bax, NF-κB, and TNF-α^100^.

Moreover, hesperidin ameliorated the hepatorenal damage caused by acetaminophen, isoniazid, rifampicin, and pyrazinamide by decreasing mRNA levels of Bax and caspase-3^101,102^. Hesperidin nearly normalized the renal function parameters, inhibited the elevated TNF-α level in the renal tissue, and attenuated the overexpression of caspase-3 and the poor expression of Nrf-2 and HO-1 in methotrexate-induced nephrotoxicity^103^. Hesperidin administration reduced apoptosis, oxidative stress, inflammation, and oxidative DNA damage significantly in sodium arsenite-induced kidney and liver tissues^41^. By upregulating Nrf2, hesperidin may help protect cardiomyocytes from the age-related increase in oxidative stress^104^. In addition, hesperidin markedly up-regulated the expression of Nrf2 and HO-1 in the liver of diethylnitrosamine /CCl_4_-induced rats^105^. Furthermore, hesperidin was found to increase Nrf2 protein expression, combating gentamicin-induced nephrotoxicity^106^, and diethylnitrosamine/CCl_4_-induced renal repercussions^107^.

We found that hesperidin administration along with CsA resulted in attenuation of cyclosporine-induced kidney injury. Hesperidin lowered the serum levels of creatinine, urea, glucose, Cyst-C, and MPO, while elevating the serum albumin; this is in line with other previous studies. Our results showed that hesperidin antagonized oxidative stress by decreasing MDA levels in kidney tissue with elevated GSH levels. Additionally, the antioxidative stress enzymes, which represent the defense mechanisms of renal SOD and CAT, were maintained near normal by hesperidin treatment. Hesperidin attenuated CsA-induced overexpression of TNF-α in kidney tissues, indicating its significant anti-inflammatory effect.

In agreement with other studies, we found hesperidin to downregulate the expression of Bax and NF-κB and upregulate the expression of Nrf-2. These findings coincide with other findings that hesperidin has cytoprotective, antioxidant, and antiapoptotic properties. It is noteworthy to mention that hesperidin ameliorated the pathological changes inhibiting the cellular infiltration within renal tissue induced by CsA and preserved the glomerular, tubular, and interstitial kidney structure, as was found previously in the mentioned studies.

Sitagliptin impedes the enzyme DPP-4, which is expressed in many tissues, including the kidneys^108^. DPP-4 inhibitors increase the circulating GLP-1 concentrations and improve glucose metabolism with antidiabetic effects^109^. Stimulation of the GLP-1R in blood vessels results in the relaxation of smooth muscle and increased renal blood flow^110^. In the normal kidney, stimulation of GLP-1R by GLP-1 results in natriuresis and water loss^111^. Treatment with DPP-4 inhibitors, which increase GLP-1 levels, has been shown to exert numerous renoprotective effects. These effects include a reduction in blood glucose and lipid levels, inhibition of inflammation and oxidative stress, amelioration of mesangial expansion, and an elevation of the glomerular filtration rate (GFR)^112^. Several mechanisms might be responsible for the renoprotective effects of sitagliptin against renal I/R such as the restoration of normoglycemia^113^.

Our results are in harmony with other studies that reported the capability of sitagliptin to improve kidney function and attenuate the histopathological changes in different experimental models such as diabetic nephropathy^114^, ischemia–reperfusion injury^34^, gentamicin nephrotoxicity^115^, and cisplatin-induced nephrotoxicity in mice^116^. In agreement with previous studies, sitagliptin produced a significant decrease in serum urea, creatinine, MPO, and cyst-C levels while preserving serum albumin compared to the nephrotoxicity group of rats.

Levels of MPO were found to be significantly higher in rats treated with CsA compared to the control group; a significant decrease in this parameter was observed in the group administered with CsA and sitagliptin or CsA and hesperidin. Catalase and SOD levels in the renal tissues were markedly decreased in the CsA-received group compared to the control animals. However, sitagliptin or hesperidin use together with CsA caused a significant increase in these antioxidant enzymes. Other studies postulated and reported the same findings^117^.

Sitagliptin exerted a renoprotective effect against ischemia–reperfusion injury through attenuating oxidative stress^118^. Moreover, sitagliptin improved cardiac mitochondrial dysfunction^119^ and attenuated brain mitochondrial dysfunction^120^ in insulin-resistant rats^115^. Oxidative stress markers such as MDA, NO, and advanced protein oxidation product concentrations were increased in the two kidney and one clip (2K1C) rats and were also normalized by sitagliptin treatment^121^.

Co-administration of Sitagliptin to gentamicin-administered rats significantly decreased the serum BUN and creatinine levels and the urinary excretion of total protein levels. Also, sitagliptin significantly restored the levels of GSH, GPx, SOD, CAT, and MDA compared to the gentamicin group^115^. Moreover, sitagliptin treatment significantly suppressed lipid peroxidation in methotrexate-intoxicated rats and significantly increased renal SOD, GPx, and catalase activities^32^. Our findings were consistent with previous work that demonstrated how sitagliptin decreased oxidative stress in the ovalbumin-induced asthma model by lowering MDA concentration and reverting SOD and GSH levels to normal^122^. Therefore, sitagliptin might reduce oxidative burden by decreasing reactive oxygen species (ROS) generation^55^.

Sitagliptin, according to Fan et al.^123^, inhibited the expression of IL-6 and TNF-α. In a rat model of heart failure, sitagliptin decreased inflammation and fibrosis since its treatment reversed the increase in TNF-α and IL-6 in the myocardium^124^. In rats with chronic cerebral hypoperfusion, sitagliptin reduced brain damage and cognitive impairment by reducing oxidative stress and the inflammatory response. Also, treatment with sitagliptin decreased the mRNA expressions of TNF-α, NF-κB, and Bax^125^. Moreover, sitagliptin was found to attenuate the renal expressions of TNF-α, and NF-κB in renal ischemia/reperfusion^34^. Additionally, sitagliptin reduced the expressions of Bax, TNF-α and NF-κB in transient cerebral ischemia/reperfusion injury in diabetic rats^126^.

Sitagliptin treatment with CsA significantly inhibited the decrease in Nrf-2 levels in the kidneys. Sitagliptin treatment in mice upregulates the Nrf2 signaling pathway and promotes Nrf2 nuclear translocation. Also, under the action of sitagliptin, Nrf2 migrated to the nucleus and inhibited autophagy, thereby reducing inflammation^127^. What’s more, sitagliptin prevented gentamicin-induced renal tubular apoptosis by exhibiting a significant decrease in caspase-3 and Bax immunoreactive cells in kidney Sections^115^.

Histologically, in streptozotocin-induced diabetic mice, there was notable renal tubular dilatation, mesangial matrix deposition, and tubulointerstitial fibrosis when compared with control mice. However, sitagliptin administration significantly ameliorated these morphologic injuries and tubular changes^128^. Sitagliptin reversed and preserved renal histostructure in different models of acute kidney injuries as well^33,112,115,116^.

Our findings demonstrated sitagliptin’s capacity to combat cyclosporine-induced nephrotoxicity. When compared to CsA-treated rats, the treatment of sitagliptin along with CsA resulted in the normalization of serum creatinine and blood urea, as well as near-normal serum albumin levels, decreased glucose levels, and decreased Cyst-C levels. The treatment of sitagliptin dramatically reduced MPO, an indication of neutrophil infiltration and activity in the kidneys. Consistent with other earlier research, sitagliptin maintained the kidney’s oxidant/antioxidant balance, as seen by lipid peroxidation inhibition, higher GSH levels, and improved SOD and CAT activity. Histopathological results of this study have shown that renal damage was successfully mitigated when animals were treated with sitagliptin before and concurrently with CsA administration. In agreement with other research, sitagliptin was found to downregulate the expression of inflammatory cytokines such as TNF-α. Also, immunohistochemical examination showed sitagliptin’s ability to impede the overexpression of Bax, which is considered an indicator of cellular apoptosis, and NF-κB, which is considered an inflammatory mediator, while on the contrary, sitagliptin upregulated the expression of Nrf-2, enhancing the antioxidant mechanisms of renal tissue.

It’s noteworthy that sitagliptin was able to preserve the structure and function of the renal tissue in rats administered CsA owing to its anti-inflammatory, antioxidant, and antiapoptotic aspects. Hesperidin and sitagliptin both exhibited almost similar effects; however, findings from biochemical, histological, and immunohistochemical studies indicated that sitagliptin would be more advantageous in avoiding kidney damage induced by CsA.

## Methods

### Animals

Mature male Wistar albino rats (n = 36), weighing 200 ± 20 g, were utilized in this investigation. They were purchased from the Animal House of the Faculty of Medicine at Assiut University, Assiut, Egypt. The animals were acclimated to laboratory conditions for 10 days, with a 12-h light and 12-h dark cycle, standard humidity, and temperature, to ensure they adapted to their new environment. Animals had free access to food and water. All experimental procedures and animal care in this study were monitored and approved by the National Research Council’s Guide for the Care and Use of Laboratory Animals and permitted by the Research Ethics Committee, Faculty of Pharmacy, Beni-Suef University (BSU-IACUC) (Number: 022-360).

### Drug and chemicals

Cyclosporine (CAS No.: 59865-13-3), Sitagliptin (CAS No.: 486460-32-6), and Hesperidin (CAS No.: 520-26-3) were purchased from Sigma-Aldrich (St. Louis, MO). All other chemicals used for the investigation were of analytical grade.

### Experimental design and animal group

**Figure 15:**
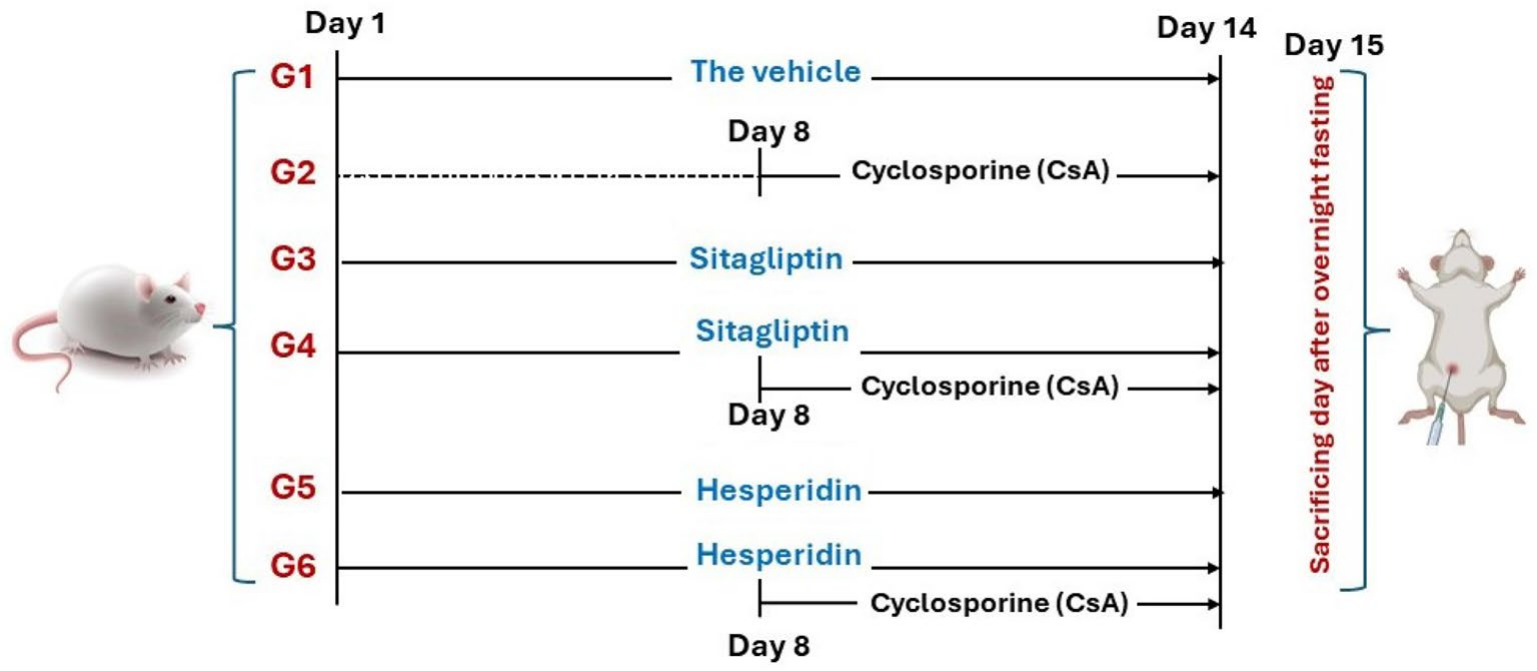
The schematic representation of the experimental design including drugs and the duration of administration. G1: Control group, G2: CsA group, G3: Sitagliptin group, G4: Sitagliptin + CsA group, G5: Hesperidin group and G6: Hesperidin + CsA group.

The animals in the present experiment were allocated into 6 groups (n = 6). Group 1 (G1): Control rats were administered to the vehicle for 14 days. Group 2 (G2): Rats received CsA (20 mg/kg/day, i.p. for 7 days)^1,129^. It was injected as a solution in normal saline for 7 days. Group 3 (G3): Rats received sitagliptin orally for 14 days at a dose of 10 mg/kg/day dissolved in saline^130,131^. Group 4 (G4): Rats received both CsA and sitagliptin regimens, as previously mentioned. Sitagliptin was administered 7 days before starting CsA, and then both drugs were given concomitantly for another 7 days. Group 5 (G5): Rats were given oral hesperidin (200 mg/kg/day) for 14 days^39,95^. Hesperidin was prepared in normal saline. Group 6 (G6): Rats received both CsA and hesperidin treatments as per the previously mentioned dose regimen. Hesperidin was administered 7 days before CsA, and for another 7 days, they were administered concurrently (Fig. 15).

Blood samples were taken under intraperitoneal pentobarbital anesthesia (35 mg/kg) from the retro-orbital plexus. Blood samples were centrifuged at 10,000 RPM for 5 min after being left for half an hour, then stored at − 20 °C until analysis. After the animals were sacrificed, the kidneys were promptly removed, and three times rinsed in ice-cooled normal saline. Each left kidney was divided into two portions; one was used for preparing kidney homogenates (10% w/v), and 500 mg of each kidney tissue was homogenized in 5 mL phosphate buffer (0.1 M, pH 7.4). The homogenates were then kept frozen at − 20 °C for the subsequent biochemical assay. The second portion of the left kidney was homogenized in RIPA buffer and utilized for western blot analysis of kidney tissue TNF-α protein expression. The right kidneys were fixed in 10% neutral buffered formalin for the assessment of histopathological renal damage and to perform immunohistochemical investigations.

### Biochemical analysis

#### Serum level of urea and creatinine

The serum levels of urea and creatinine were determined using available commercial kits [COD 11,516 (urea), COD 11,734 (creatinine), BioSystems S.A. Costa Brava, Barcelona, Spain] and according to the instructions of the manufacturer.

#### Serum albumin and cystatin C

By using a commercially available ELISA kit (Cat. No. BSIS02-I), the serum level of albumin was measured following the instructions of the manufacturer (SPINREACT, Santa Coloma, Spain). The serum level of cystatin-C was estimated using a commercially available Rat Cys-C ELISA kit (Catalog No.: E-EL-R0304) and following the manufacturer’s instructions (Elabscience Biotechnology Co., Ltd., Wuhan, Hubei, China).

#### Determination of serum myeloperoxidase (MPO)

According to the manufacturer’s instructions, serum MPO was determined using the available ELISA kit from FineTest Biotech Inc., US (Cat. No.: ER0142).

#### Blood glucose level

It was performed using an available commercial kit (Cat. No. BSIS17-1), and the serum level of albumin was measured following the instructions of the manufacturer (SPINREACT, Santa Coloma, Spain).

#### Determination of tissue malondialdehyde (MDA)

The renal tissue level of MDA was estimated as an indicator of lipid peroxidation. This was performed according to instructions and steps described in the colorimetric method described by Ohkawa et al.^132^ and by using a commercially available kit (CAT. No. MD 2529) (Biodiagnostic, Giza, Egypt).

### Determination of glutathione reduced (GSH)

According to the method described by Beutler et al.^133^ the GSH level in the kidney tissue was using a commercially available kit (CAT. No.: GR 2511) (Biodiagnostic, Giza, Egypt). The method is based on the reduction of 5,5′ dithiobis (2-nitrobenzoic acid) (DTNB) with glutathione (GSH) to produce a yellow compound. The reduced chromogen is directly proportional to GSH concentration, and its absorbance can be measured at 405 nm.

#### Determination of catalase (CAT) activity

This was done by the utilization of an available colorimetric kit (CAT. No.: CA 2517, Biodiagnostic, Giza, Egypt) and a method described by Aebi^134^.

#### The SOD activity

The principle of the total SOD activity method is based, briefly, on the inhibition of nitro blue tetrazolium (NBT) reduction by the generated xanthine/xanthine oxidase system^135^. The activity was assessed in the ethanol phase of the serum after the addition of 1.0 ml of the ethanol/chloroform mixture (5/3, v/v) to the same volume of centrifuge. One unit of SOD was defined as the enzyme amount causing 50% inhibition in the NBT reduction rate. The SOD activity was expressed as a U/g protein. The assay was performed using a commercially available colorimetric kit (CAT. No. SD 2521, Biodiagnostic, Giza, Egypt).

#### Western blot analysis for renal TNF-α

To achieve a clear supernatant, tissue samples were centrifuged after being homogenized in RIPA buffer. The total protein content was determined using the Bradford reagent. SDS-PAGE was used to isolate 30 µg of protein per gel lane, and the protein was then transferred to a PVDF membrane. The membranes were then treated with primary antibodies against TNF-alpha antibody after being blocked in Tris-buffered saline with Tween 20 (TBST) containing 5% non-fat milk powder (Novus Biologicals USA). By ensuring that protein loading is uniform throughout the gel, the housekeeping protein β-actin was utilized as a loading control to normalize the quantities of protein observed. The membranes were TBST-washed before being incubated for an hour with horseradish peroxidase-conjugated secondary antibodies from Novus Biologicals in Littleton, Colorado, USA. An improved chemiluminescence kit was used to find immuno-labeling (BioRad, Hercules, CA). Finally, we will utilize ImageJ to scan the acquired blots and quantify band intensities (NIH, Bethesda, Maryland, USA).

#### Histopathological examination

The obtained kidney specimens from each rat were fixed in the following fixative: 40 mL paraformaldehyde, 125 mL phosphate buffer (0.2 M, pH 7.4), 25% freshly made, 37.5 mL saturated picric acid, 0.5 mg calcium chloride, 1.25 mL glutaraldehyde 25%, and distilled water was added up to 250 mL. The specimens were fixed using Wrobel–Moustafa fixative for 24 h. The fixed samples were thoroughly washed with 70% ethanol three times over 24 h. Following cleaning, samples were encased in paraffin wax. Using a Reichert Leica RM2125 Microtome, sections were cut between 5 and 7 µm.

Hematoxylin and eosin stain was used to stain paraffin representative sections for general histological study and scoring (H&E). Other sections were stained with Periodic Acid-Schiff (PAS) stain to evaluate the glomerular basement membrane thickening and damage. Additionally, Sirius red stain was used to investigate fibrosis (Red color) in the kidney section of various groups^136,137^.

Sirius Red staining was performed to determine the percentage fibrosis area: incubating slides in 0.1% Sirius Red F3B for 1 h, washing twice in acidified water, dehydrating thrice in 100% ethanol, and then clearing in xylene Interstitial fibrosis is commonly measured by histology. Sirius Red staining is specific for collagen type I and III fibrils^138^. Sirius red fibrosis was quantified using image analysis to determine the percentage fibrosis area^136^.

According to the method published by Abd-Eldayem et al.^139^ we used Image J to calculate the area % of staining intensity of PAS stain in images from various experimental groups. Launch the image editing software J Fiji and open each picture separately. Click “type” and then “8-bit” to convert the image in the image column to an 8-bit image; click “analyze column” and choose the measurement; click “okay” after verifying the area and area fraction; click “image” and click “adjust” and then click “threshold” You can change the backdrop color to white, red, or black using the drop-down choices. Adjust the top slider until the foreground of the image is fully red to perform a threshold. As much as possible, maintain a uniform stain. To finish, hit the “Apply” button.

### Immunohistochemical determination of Bax and Nrf-2 and NF-κB

Paraffin tissue sections were rehydrated and deparaffinized using ethanol and double-distilled water. Antigen retrieval was performed as slides were completely submerged in excess amounts of a pre-heated antigen retrieval solution and microwaved until boiling. Continuous boiling was maintained for at least 15 min. After boiling, we washed slides four times in buffer, followed by the application of block, and then incubated for 5 min at room temperature and away from light. Then, they were washed once in the buffer. After washing, the primary antibodies for Bax [Bax antibody (Cat. No. GTX32465), Gene Tex, USA], Nrf2 [NRF2 antibody (Cat. No. GTX55732), Gene Tex, USA], and NF-κB [NFκB p105 (phospho Ser927) antibody, (Cat. No. GTX32222), Gene Tex, USA] were applied at a dilution of 1/100 and incubated overnight. Washing was done four times in the buffer, followed by placing the biotinylated link antibody, and then left at room temperature for 15–20 min. After washing four times in the buffer, streptavidin/HRP was applied and then left at room temperature for 20 min. Following four times rinsing in the buffer, DAB chromogen was mixed thoroughly and then applied to slides for 5 min. After rinsing once in distilled water. the DAB Chromogen/Substrate was applied for 5 min before rinsing and this was followed by counterstaining. The coverslip was placed for each slide using DI followed by examination. We utilized a Leitz Dialux 20 microscope and a Canon PowerShot A95 digital camera to evaluate immunohistochemistry staining.

### Statistical analysis

Statistical assessment was conducted using GraphPad Prism 8 software (One-way ANOVA with a post-hoc Tukey’s correction) and the SPSS program (version 17) (One-way ANOVA, followed by the Scheffe and Duncan test). To identify any significant differences between the pre-treatment and treatment groups, a T-test was applied. A *p*-value < 0.05 was deemed significant.

### Ethical approval and consent to participate

The study’s protocol was approved by the Faculty of Pharmacy, Beni-Suef University’s Ethics Committee for Animal Experimentation (BSU-IACUC) (Number: 022-360). All methods were performed in accordance with the relevant guidelines and regulations. The study is also presented in accordance with ARRIVE guidelines.

### Supplementary Information

Supplementary Information.

## Data Availability

The datasets generated and/or analyzed during the current study are available from the corresponding author upon reasonable request.
